# A multi-tissue type genome-scale metabolic network for analysis of whole-body systems physiology

**DOI:** 10.1186/1752-0509-5-180

**Published:** 2011-10-31

**Authors:** Aarash Bordbar, Adam M Feist, Renata Usaite-Black, Joseph Woodcock, Bernhard O Palsson, Iman Famili

**Affiliations:** 1 GT Life Sciences, 10520 Wateridge Circle, San Diego, CA 92121, USA; 2 Current Affiliation: Intrexon Corporation, 1872 Pratt Dr., Blacksburg, VA 24060, USA; 3 Current Affiliation: Department of Bioengineering, University of California San Diego, 417 Powell-Focht Bioengineering Hall, 9500 Gilman Dr., La Jolla, CA, 92093-0412, USA; 4 Genomatica, 10520 Wateridge Circle, San Diego, CA 92121, USA

## Abstract

**Background:**

Genome-scale metabolic reconstructions provide a biologically meaningful mechanistic basis for the genotype-phenotype relationship. The global human metabolic network, termed Recon 1, has recently been reconstructed allowing the systems analysis of human metabolic physiology and pathology. Utilizing high-throughput data, Recon 1 has recently been tailored to different cells and tissues, including the liver, kidney, brain, and alveolar macrophage. These models have shown utility in the study of systems medicine. However, no integrated analysis between human tissues has been done.

**Results:**

To describe tissue-specific functions, Recon 1 was tailored to describe metabolism in three human cells: adipocytes, hepatocytes, and myocytes. These cell-specific networks were manually curated and validated based on known cellular metabolic functions. To study intercellular interactions, a novel multi-tissue type modeling approach was developed to integrate the metabolic functions for the three cell types, and subsequently used to simulate known integrated metabolic cycles. In addition, the multi-tissue model was used to study diabetes: a pathology with systemic properties. High-throughput data was integrated with the network to determine differential metabolic activity between obese and type II obese gastric bypass patients in a whole-body context.

**Conclusion:**

The multi-tissue type modeling approach presented provides a platform to study integrated metabolic states. As more cell and tissue-specific models are released, it is critical to develop a framework in which to study their interdependencies.

## Background

Metabolism has been implicated in most major human diseases including obesity, diabetes, cancer, and heart disease [1]. Thus, metabolism has been a field of study integral to many branches of medicine. In particular, obesity, a leading preventable cause of death, increases the likelihood of heart disease and diabetes, and represents one of the most serious current public health care problems [2]. Studying and understanding such systemic diseases however requires a fundamental and comprehensive analysis of not only the individual tissues and cell types, but also their integrated functions and interlinked interactions. Accurate physiological representation and analysis of systemic diseases however cannot be achieved unless an integrated multilevel and comprehensive modeling approach is undertaken and the appropriate computational infrastructure is fully developed and utilized.

Genome-scale metabolic network reconstructions have been shown to provide an appropriate context for analyzing biological content [3]. Metabolic reconstructions are important for elucidating the genotype to phenotype relationship and have proved to be useful in interpreting high-throughput, omic data sets [4]. Genome-scale networks can account for a combination of genetic and physiological data [3]. Metabolic networks are built in a bottom-up approach, consisting of the known genes, transcripts, proteins, reactions, and metabolites. Recently, a global human metabolic network (Recon 1) was reconstructed [5]. Recon 1 is a comprehensive map of all the known annotated metabolic reactions of human cells, containing 1496 genes, 3402 intracellular reactions, and 2785 metabolites.

Recon 1 was developed to provide a global genome-scale description of human metabolic capabilities, without consideration of tissue-specific information. As particular cells in the human body do not use all the metabolic capabilities encoded on the genome, procedures have been developed to tailor Recon 1 to tissue and cell specific functions [6-8]. Models for the human liver [8,9], kidney [10], brain [11], erythrocyte [12], and alveolar macrophage [13] have been developed. To study diabetes in obese individuals, three tissue-specific reconstructions of the liver (hepatocyte), skeletal muscle (myocyte), and adipose tissue (adipocyte) were developed in this study. The liver plays the biggest role in metabolism, with many functions including gluconeogenesis, glycogen storage, urea production and ketogenesis. Though the liver consists of many different cell types, the major cell type pertaining to metabolism is the hepatocyte. Skeletal muscle, one of the most abundant tissues in the human body, has high metabolic requirements, and plays a major role in protein metabolism and storage. Adipose tissue plays a major role in lipid metabolism and storage. Skeletal muscle and adipose tissue primarily consist of myocytes and adipocytes, respectively.

**Figure 1:**
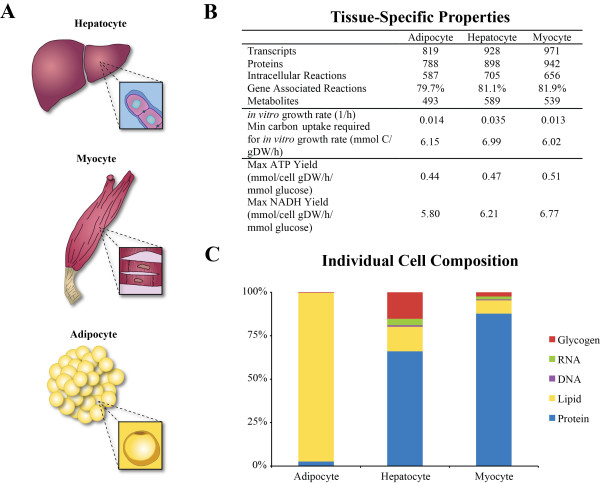
**General properties and characteristics of individual cell-specific metabolic reconstructions**. **(A) **We modeled three cell-specific metabolic networks of human tissues: hepatocyte from liver, myocyte from skeletal muscle, and adipocyte of adipose tissue. **(B) **The tissue specific properties are broken up into three main sections. The first describes the topological and knowledge base characteristics of the metabolic networks. The second shows the *in vitro *growth rate and the required non-glucose carbon amount to maintain that growth rate. This growth rate was used as a maintenance function to model biological turnover. The third section details the energy and oxidative capacities of the networks. Note that the growth rate was set as a constraint for these simulations. **(C) **The biomass maintenance functions for the three metabolic networks were built based on the individual dry cell weight compositions. The AM is primarily composed of lipids while the MM is of protein. The HM has a more balanced composition with protein, glycogen, and lipids being the major components.

To study the metabolic interdependence within the human body, a novel multi-tissue modeling approach was developed to combine three cell type specific metabolic reconstructions (see, Figure 1A). Using gene expression data and constraint-based analysis methods [14], the metabolic differences in obese and obese type II diabetic individuals were studied and the difference of activity in metabolic reactions was investigated between the two groups.

## Results and Discussion

To clearly convey the characteristics and capabilities of the three cell-specific reconstructions and the integrated multi-tissue type network, the results presented here are split into three distinctive sections.

• The reconstruction, the content, the quality controls, and the properties of the individual hepatocyte, myocyte, and adipocyte metabolic networks (designated as HM, MM, AM, respectively) are detailed.

• The process of building the integrated multi-tissue type network is described and the integrated network is used to compute and compare three established metabolic states (i.e., the Cori cycle, the Alanine cycle, and the absorptive or post-feeding state).

• Expression-profiling data was utilized to build two context-specific multi-tissue type models detailing metabolism in i) obese vs ii) diabetic obese individuals to ascertain their differences.

### The content of the cell-specific metabolic network reconstruction

**Figure 2:**
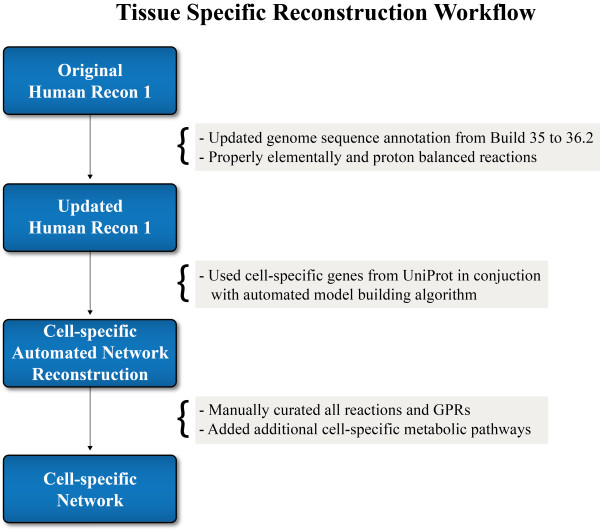
**Workflow for building the cell-specific reconstructions**. Human Recon 1 [5] was utilized as the starting point for modeling human metabolism. The genome sequence annotation upon which Recon 1 was built was updated, and all incorrectly lumped and balanced reactions were corrected. Multiple reaction and enzyme databases, including UniProt, were then used to build a draft model. The draft model was then manually curated using literature and multiple high-throughput data sets. In addition, important metabolic pathways that were not in Recon 1 (e.g. hepatic ketogenesis) were added accordingly to the cell-specific reconstructions.

Three human cell-specific metabolic networks were reconstructed for three major tissue types: liver (hepatocyte), fat (adipocyte), and skeletal muscle (myocyte). The overall metabolic network reconstruction workflow used to establish the three networks is depicted in Figure 2. First, the human genome sequence and annotation was updated in the SimPheny™ modeling platform (Genomatica, Inc.) from Build 35 to Build 36.2. Utilizing tissue-specificity information from UniProt, Recon 1 was filtered to draft network reconstructions of the human hepatocyte, adipocyte, and myocyte. The draft reconstructions were then manually curated and augmented with additional information taken from online databases and published literature. Unlike current automated cell-specific models that are based solely on Recon 1, significant changes were made to build more accurate and quality controlled network content for the human hepatocyte, adipocyte, and myocyte metabolism. This curation and validation process included the followings:

• The models were tested for internal pathway cycles that could generate excess energy when all exchanges were closed. Appropriate changes were made to the model to remove all futile cycles.

• All extraneous dead end and gap-containing pathways were removed to build fully functional networks.

• All reactions were elementally and charge balanced and none of the metabolic pathways were lumped into single reactions.

• Published data for the different tissues was used to define biomass objective functions (see Additional File 1). The biomass functions were used as maintenance lower bounds for all cell-specific and multi-tissue type simulations.

A detailed description of the cell-network reconstruction pipeline, manual curation, removal of futile cycles, and biomass definition is provided in the Methods section. In general, the processes for generating quality controlled and functional reconstructions of metabolism are well established and available elsewhere [15,16]. The workflows used to generate the multi-tissue type model adhere to these established procedures.

### Global and cell-specific validation using network models for assessing functional states

In order to validate the three cell-specific networks, universal testing was completed for quality control and assurance purposes. Universal testing consisted of validating general metabolic functions of the human cells. The ability to produce precursor metabolites of major metabolic pathways from glucose was confirmed. Production of the nine non-essential amino acids, two conditionally essential amino acids, the fatty acids, nucleotides, glycogen, and cholesterol was also validated. Finally, the three cell-specific models were checked for production of biomass, representing cell growth.

After global testing, cell-specific functions were tested. Cell-specific lipid production of each of the metabolic models was validated. The hepatocyte has many metabolic functions such as gluconeogenesis, ketogenesis, urea and bile production, and glycogen storage. For the HM, we validated that the metabolic network could synthesize glucose from gluconeogenic substrates, such as amino acids, glycerol, lactate, and pyruvate. The pathways for ketogenesis were not included in Recon 1, but were added to the HM to produce ketone bodies. The HM's ability to use alternative sugars as energy sources, complete the urea cycle, and process nitrogen to produce urea was also validated. Fat cells store energy as triglycerides and other lipids. For the AM, the cell-specific model's ability to generate lactate, glycerol, and fatty acids from glucose and triacylglycerol was validated. Finally, skeletal muscle facilitates movement in the body by converting chemical energy into mechanical energy. For the MM, the network's ability to produce ATP from glucose, branched chain amino acids, ketone bodies, glycogen, and fatty acids was validated. In addition, a cell-specific objective function was constructed that utilizes ATP to model the metabolic toll of muscle contraction.

The final cell-specific reconstructions and mathematical models and a full breakdown of every universal and cell-specific test is provided in the Supplementary Material (see Additional Files 2, 3, 4).

### Characterizing the cell-specific networks

After an extensive QC/QA procedure, the three cell-specific metabolic networks were characterized and tested for network capabilities (Figure 1B). While the models contain a relatively similar number of metabolites and reactions, the HM has the largest model among all three. This difference is due to the HM's diverse metabolic capabilities that has been captured in the model. For all simulations, the biomass function of each of the metabolic models was set to a physiologically relevant lower bound determined from the cells' *in vitro *growth rate [17-19]. A lower bound was set in order to simulate regular cellular maintenance such as protein, mRNA, and lipid turnover as well as DNA repair and ATP maintenance requirements. To examine the models, the minimum required amount of carbon was calculated for each of the models to match its *in vitro *growth rate. This value shows the metabolic requirement for each of the individual cells to be viable. The HM has a much greater need for carbon than the other networks to maintain the *in vitro *growth rate (Figure 1B). This greater need is not due solely to the higher set growth rate. Though the lower bound of the HM's growth rate is set roughly two and a half times higher than the AM's, the HM's non-glucose carbon uptake is almost seven times as much showing a higher varied metabolic need from the extra-system to function.

**Figure 3:**
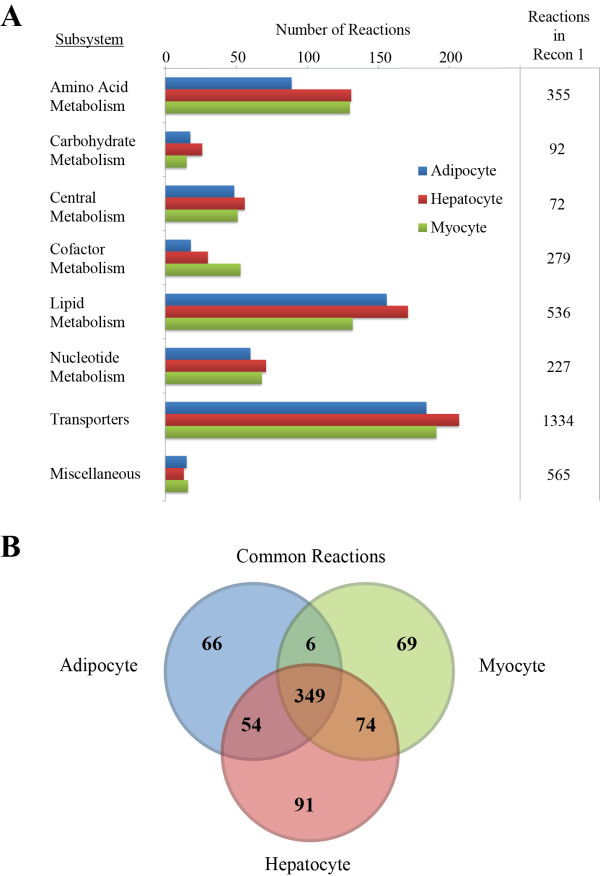
**Comparison of the metabolic reactions in the three cell-specific reactions**. **(A) **The three cell-specific reconstructions have a similar number of reactions in most subsystems. It is interesting to note that the AM has much fewer amino acid metabolic reactions and the MM fewer lipid metabolic reactions. The reaction number difference is in accordance with the cell composition differences. The HM has the most reactions in most subsystems due to the liver's varied metabolic states. For comparison, the number of reactions in each subsystem is also provided for Recon 1. **(B) **The three cell-specific reconstructions share the most intracellular metabolic reactions that seem to be a "core" group of reactions required for most human cells. The rest of the reactions are mostly exclusive. The AM and MM have very little reaction similarity that is not also found in the HM.

The metabolic capabilities of the three networks were further tested. The three cell-specific models produce approximately the same maximum ATP and NADH yields, with the MM producing the most. The MM's ability to produce more cofactors is probably due to its higher number of metabolic reactions dealing with cofactor metabolism (Figure 3A). Finally, after constructing the biomass functions from the dry cell weight compositions, there was a rather varied metabolic composition for each of the three cells (Figure 1C). As expected, the AM biomass consists mainly of lipids, the MM consists mainly of protein, and the HM has a more heterogeneous composition with the majority being protein.

### Comparing the cell-specific networks

The three cell-specific reconstructions were also compared based on the assigned subsystems and reactions. The reactions of each model were grouped into subsystems (Figure 3A). The majority of reactions in all of the networks corresponded to transporters and lipid metabolism. As the AM has the least metabolic variation and the smallest network, it has the lowest number of reactions in most subsystems except for lipid metabolism. The hepatocyte is the most metabolically active and performs the most metabolic functions of the three cell types. Hence, the HM has the most reactions in each subsystem except for cofactor metabolism. The MM has the most reactions corresponding to cofactor metabolism, due to the skeletal myocyte's high conversion of cofactors for producing chemical energy. Compared to Recon 1, which consists of 3,311 metabolic and transporter reactions, the three cell-specific models have much fewer reactions. The network content and functional scope captured in the cell-specific models reflects specific tissue function with focus on developing high quality metabolic models, as compared with comprehensive but less functional metabolic maps. The three cell-specific reconstructions share a core network of 349 reactions (Figure 3B), which represents the majority of the metabolic reactions in all of the models. Of the remaining reactions, the majority is unique to each cell-specific model. The HM shares a substantial number of reactions with the AM and MM (54 and 74 respectively), while the AM and MM share only six reactions that are not in the HM. Thus, as expected, the three cell-specific metabolic models have functional metabolic uniqueness, but require the same core reactions for basal functionality.

### Network integration: connecting the cell-specific reconstructions through a blood compartment

After validation and characterization of the metabolic models on an individual level, the next step was to simulate the integrated metabolic function of all three cell-types. As the three cell-types represent the most important metabolically active cells of their respective tissues, the integrated modeling and simulations are termed multi-tissue modeling and simulation.

**Figure 4:**
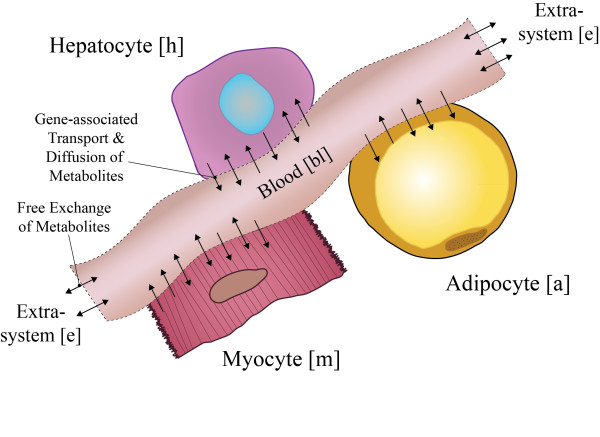
**Schematic of the multi-tissue modeling approach**. The three cell-specific reconstructions are combined into a multi-tissue model by connecting them all to a new blood compartment. Metabolites enter the model through the extra-system through exchange reactions. Metabolites are then imported into the different cells through gene associated intercellular transporters and/or free diffusion. For differentiating the cell-specific models, all reactions in the model were annotated with [a], [h], [m], and [bl] for the AM, HM, MM and blood compartment, respectively.

In order to connect the three reconstructions, a new blood compartment was created to simulate transport functions of the blood, with the ability of metabolites to leave the blood for physiological processes such as renal clearance. Network exchanges with the extra-system were facilitated through the blood. The three cell type models imported or secreted metabolites into the blood through gene-associated transporters and diffusion, when appropriate (Figure 4). Though the process seemed straightforward, there was difficulty in computing a steady-state for the integrated network. Interestingly, it turns out that the intercellular transport of metabolites is not properly proton balanced. A bicarbonate buffer system was added to the integrated model in the blood compartment to balance protons. The buffer reaction was not initially thought to be necessary until the requirement for balancing the protons in the interstitial space became apparent during network simulations. This requirement is consistent with the underlying human physiology [20]. Thus, the multi-tissue model is more than strictly a network addition of three single cell models. The addition of the buffer system is neither intuitive nor obvious, though its physiological role was clearly relevant and became apparent in multi-tissue metabolic modeling.

### Forming a multi-tissue model

**Figure 5:**
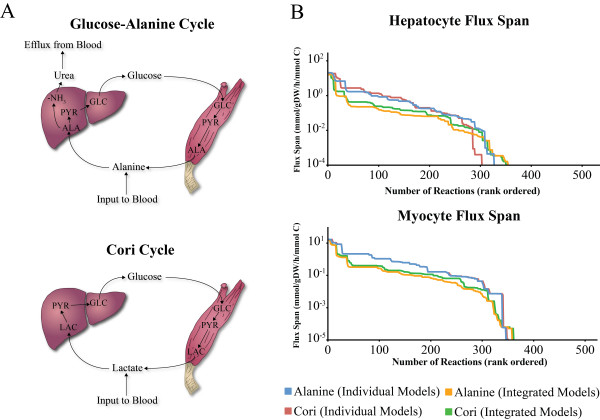
**The Alanine and Cori cycles of human metabolism**. **(A) **The Alanine and Cori cycles are methods for peripheral tissues to receive glucose under nutrient limited situations. Gluconeogenic substrates (e.g. alanine and lactate) are released from peripheral tissues and absorbed by the liver to produce glucose. The glucose is then returned to the peripheral tissues for their metabolic requirements. **(B) **The flux spans for the HM and MM under individual or integrated simulations are shown. The multi-tissue modeling approach has a constraining effect on the HM and MM models (see Table 1). GLC = glucose, ALA = alanine, PYR = pyruvate, LAC = lactate.

**Figure 6:**
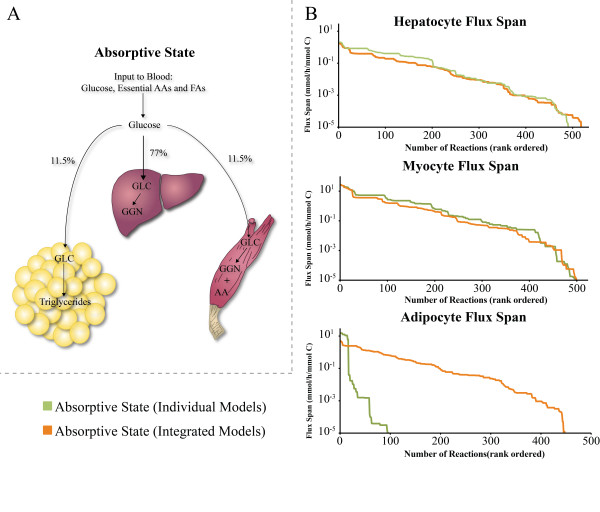
**The absorptive state of human metabolism**. **(A) **In the absorptive state, food is digested and absorbed primarily as glucose and amino acids. The schematic shows influx of glucose into the blood and the modeling-defined fractions of intake into each of the three models. Essential amino acids and fatty acids were also provided. For the multi-tissue simulations, the AM model stores triacylglycerol, the MM stores protein and glycogen, and the HM stores glycogen. Some of the glucose delivered to the HM was converted to fatty acids that are transported to the AM for triacylglycerol production. **(B) **The flux spans for the three cell-specific reconstructions individually and when integrated are shown. Integration had a constraining effect on the HM and MM models, but had an opposite effect on the AM. This was due to fatty acid production by the HM that was then transported to the AM. GLC = glucose, GGN = glycogen, AA = amino acids, FAs = fatty acids.

The integrated multi-tissue metabolic model was used to simulate three physiologically important metabolic states in the human body: the Alanine cycle, Cori cycle, and absorptive state (Figures 5 and 6). The flux span of the networks (see Methods) was determined to show the metabolic differences between the cycles and the individual cell-specific models. The flux span provides insight on the flexibility of the network as well as the overall size of the solution space. It is important to note that without proper *in vivo *isotopomer flux measurements, all results are qualitative. However, these three examples illustrate the potential of multi-tissue metabolic models.

As the inputs are relative rather than absolute, the fluxes were normalized to the amount of carbon input. Using this reference point, the percentages of uptakes and secretions presented below demonstrate the physiological behavior. In addition, because the liver, adipose tissue, and skeletal muscle have different masses, the canonical units for genome-scale reconstructions, mmol/h/g cell DW, cannot be used. Using the g cell DW portion of the units assumes that reactions in all three tissues have the same mass. Such an assumption would skew the intercellular fluxes. In order to account for the mass discrepancy in different tissues, the units were changed to mmol/h/body by modifying the biomass objective functions to represent the entire maintenance of each tissue (see Methods).

The Alanine and Cori cycles are physiologically relevant metabolic cycles, but they do not occur exclusively physiologically. In order to amplify and study each one separately, constraints were set on the MM's ability to produce either alanine or lactate and hence affect HM substrate utilization. An unconstrained assessment of MM production and HM use of alanine and lactate to produce glucose and urea in the liver is presented in the Supplementary Material (Additional File 5).

### Metabolic state 1: the Alanine cycle

The Alanine cycle is an important physiological cycle that occurs between the myocyte and hepatocyte under glucose limiting conditions [21]. The cycle's function is to eliminate nitrogen from the myocyte and transport it to the hepatocyte for degradation as urea in exchange for energy in the form of glucose. In the liver, alanine is deaminated into pyruvate, which serves as a substrate for gluconeogenesis. In return, glucose is supplied from the liver to skeletal muscle. The multi-tissue simulation consists of the HM, MM, and the blood compartment. In this condition, alanine was imported from the extra-system, i.e., the blood stream (Figure 5A). The HM imports alanine and produces both glucose and urea. The carbon conversion is not one-to-one due to the maintenance requirements of the HM. The carbon split becomes 47:18:35 for glucose, urea, and cellular maintenance requirements, respectively. Taking into account the cellular maintenance requirements provides a more realistic carbon conversion of the Alanine cycle.

The flux spans of the individual HM and MM were investigated and compared to the multi-tissue simulation (Figure 5). For the individual models, the same multi-tissue simulation setup was used but all reactions in the other cell types were inactivated. It became immediately apparent that the two cells metabolically interact with one another. For the Alanine and Cori cycle simulations, because carbon is recycled between the liver and muscle, the extra-system exchanges were not open for carbon sources to exit the blood, thus the individual cell-type models simulations yielded only infeasible solutions. When simulating the individual cell-type models, the exchanges with the extra-system had to be opened for a proper mass balanced steady-state solution.

In contrast, the multi-tissue simulation finds a mass-balanced steady state by exchanging metabolites between the HM and MM. Combining the two models had two major effects. First, since the HM and MM became dependent on each other metabolically, the models constrained each other, shown by a lower mean flux span and higher number of fixed fluxes (Figure 5 and Table 1). A fixed flux has equivalent minimum and maximum optimized values. A multi-tissue model thus can properly simulate a physiological cycle and show intercellular interactions that an individual cell model cannot. Second, the number of fixed zero fluxes decreased in the multi-tissue simulations (Table 1). The HM and MM are linked, allowing for one cellular model to act as another's sink or source. More metabolic pathways can be potentially active in a mass balanced steady-state solution, making the multi-tissue models more robust in a nutrient limited state.

**Table 1:** Flux span and number of reactions participating in internal loops and carrying zero and fixed fluxes of individual and multi-tissue models

Cell Type	Mean Non-Zero Flux Span (mmol/h/body/ mmol C)	Number of Reactions in Loops	Number of Zero Flux Reactions	Number of Fixed Flux Reactions
Individual HM (Alanine)	0.141	38	217	111
Individual MM (Alanine)	0.149	43	188	63

Individual HM (Cori)	0.172	38	225	125
Individual MM (Cori)	0.153	43	186	64

Individual AM (Absorptive)	0.044	22	54	15
Individual HM (Absorptive)	0.13	38	123	0
Individual MM (Absorptive)	0.18	40	100	0

HM (Alanine)	0.0588	38	178	111
MM (Alanine)	0.0619	43	143	100

HM (Cori)	0.0543	38	178	111
MM (Cori)	0.0712	43	141	100

AM (Absorptive)	0.062	22	59	99
HM (Absorptive)	0.070	38	125	94
MM (Absorptive)	0.13	40	99	51

### Metabolic state 2: the Cori cycle

The Cori cycle is a metabolic cycle, similar to the Alanine cycle, that metabolically connects the peripheral tissues with the liver [21]. Lactate acts as substrate for hepatic gluconeogenesis. As with alanine in the Alanine cycle, lactate is taken up by the liver and is converted into pyruvate, in this case by lactate dehydrogenase. Unlike the Alanine cycle, no major byproducts (e.g. urea) are generated, and thus the Cori cycle is a cycle of energy transfer between two tissues. The Cori cycle simulation involves the HM, MM, and blood compartment. It is set up with an input of lactate into the blood from the extra-system (Figure 5B). The Cori cycle's efficiency was validated using flux balance analysis of the integrated multi-tissue model. The HM takes up lactate and converts it into glucose. The remainder of the carbon is used for cell maintenance purposes, as described earlier. The carbon split for this cycle is 57:43 for glucose and maintenance respectively. Lacking a byproduct, the cycle converts about 10% more carbon into glucose for the peripheral tissues than the Alanine cycle.

As before, the flux span of the multi-tissue simulation and individual cell-type models was compared (Figure 5). The approach was used as before with similar results. The solution space had shrunk, and the number of zero fluxes had dropped in the multi-tissue simulation. The similarity in results is due to the fact that the two cycles having similar physiological purposes. The HM takes up a gluconeogenic substrate and produces glucose for the MM in a glucose-poor environment.

### Metabolic state 3: the absorptive state

The final multi-tissue simulation presented, the absorptive state, is physiologically different than two metabolic cycles already discussed. Thus different results were obtained when investigating its flux span. The absorptive state is an anabolic process during which absorbed glucose is used by the human body to produce glycogen, triaclglyerol, and amino acids [20]. Metabolic function of the liver during the absorptive state is closely linked to the adipose tissue and skeletal muscle for energy storage (Figure 6A).

During the absorptive state, carbohydrates and proteins in food are primarily absorbed as monosaccharides (i.e., essentially glucose) and amino acids. The liver absorbs a fraction of the blood glucose and the rest is taken up by peripheral tissues in the body to generate ATP for energy maintenance requirements. In addition to energy generation, the absorbed glucose is stored as triacylglycerol in the adipose tissue and as glycogen and proteins in the muscle tissue. The excess glucose in the liver is similarly stored as glycogen and triacylglycerol, however unlike the adipocyte, only a small amount of the synthesized triacylglycerol is stored in the liver and the rest is transported to adipose tissue.

An absorptive multi-tissue simulation was performed and consisted of the three cell types, HM, MM, AM, and blood compartment connecting them. Nutrients were absorbed from the extra-system entering the blood compartment. Absolute values for glucose uptake rates were taken from literature [22]. *In vivo *amino acid levels in the blood are elevated between two- to seven-fold during the absorptive state [23]. Assuming that the minimum requirement of amino acids for biomass maintenance is the baseline level in the blood, the amino acid influx was scaled between two to seven times depending on the specific amino acid. Fatty acids were similarly scaled, in the absence of specific data.

Due to the many metabolic objectives of the absorptive state, for simulation, a Pareto optimality approach was used [24]. Briefly, this optimization framework involves optimizing for a specific objective, then fixing that flux, then optimizing for another objective. If there are more than two objectives, the process is repeated. First, AM triacylglycerol production was optimized and the resulting flux was fixed. Then, a baseline level of amino acid production by the MM was set. The amino acid lower bound for the MM was scaled to a ratio similar to the MM biomass amino acid makeup. Finally, concurrent production of glycogen in the HM and MM was optimized.

When comparing the multi-tissue absorptive flux span with the individual models in similar conditions, there were some differences as compared to the Cori and Alanine cycles (Figure 6B). Initially, the individual AM flux span could not be calculated due to its inability to produce as much triacylglycerol as the set lower bound for the multi-tissue simulation. The glucose uptake of the AM was set from a physiological constraint [22], limiting triacylglycerol production. To reach the triacylglycerol production lower bound, the HM converts some of its glucose to triacylglycerol precursors that are then transported to the AM, very similar to what occurs physiologically [20]. Because the multi-tissue simulation does not have a triacylglycerol lipoprotein transporter, the fatty acid precursors were formed by the HM and transported to the AM for maximal triacylglycerol production. In the individual AM, fatty acid uptake was increased to complete the study. The HM providing fatty acids to the AM for triacylglycerol has two consequences on the solution space: 1) the multi-tissue HM's solution space is highly constrained compared to that of the HM for the individual hepatic cell-type, while 2) the multi-tissue AM solution space is much larger than for the individual cell-type AM. Despite these differences, on the whole, the mean flux span of all three networks is reduced. There were also a larger number of fixed fluxes in the multi-tissue simulation, due to the higher interdependence between the three cell-specific models. The absorptive state is not limiting and all input metabolites were provided in excess. When simulating the individual models, there were very few to no fixed fluxes in the individual models due to the non-limiting constraints.

### Recap of results for the three multi-tissue metabolic state computations

Three physiologically relevant cycles were simulated using the multi-tissue approach. The flux span computations yielded two main results: 1) the intracellular dependence between different cell types in a multi-cellular organism and 2) the transfer of fatty acids to the AM from the HM. Multi-tissue models can provide a platform for mapping isotopomer flux measurements to further increase quantitative accuracy of physiological cycles and holistically understand human metabolism.

### Metabolic Differences in Obese and Diabetic Obese Individuals

A major application of genome-scale reconstructions is providing a systems context for integrating high-throughput data, also known as "context for content" [25]. Transcriptomics and proteomics can be appropriately mapped onto the reactions of metabolic networks to allow for a systems analysis of the data.

The metabolic differences between i) obese and ii) Type II diabetes obese individuals were analyzed using the multi-tissue type model developed above. Transcriptomic data was obtained from adipose, liver, and skeletal muscle tissue samples of gastric bypass surgery patients in a fasting state (see Methods). In order to properly simulate the flux conditions, a baseline metabolic state was established. The absorptive state was adapted to simulate a prolonged starvation state. To perform this analysis, the objectives of the absorptive state (AM triaclyglycerol production and MM protein production) were converted into the inputs of the system and the objective was set as the HM glucose production. The changes in the multi-tissue simulation provide a functional backdrop to analyze the obese and Type II diabetes obese states.

**Figure 7:**
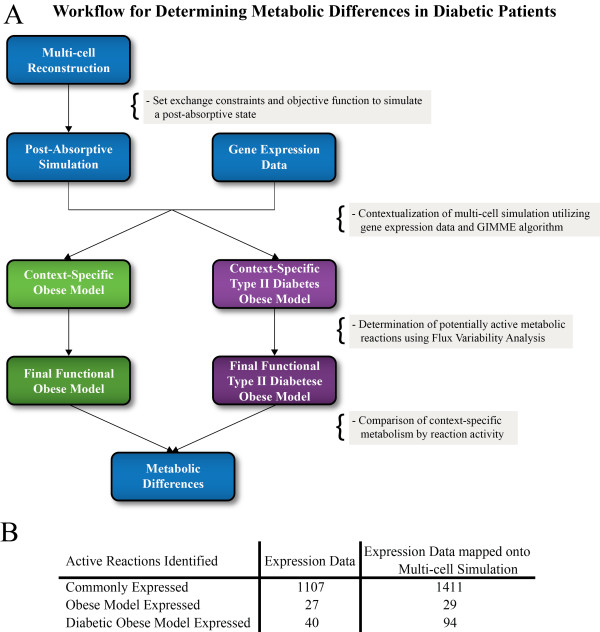
**Workflow and characteristics of context-specific models**. **(A) **Two context-specific multi-tissue metabolic networks were built using post-absorptive exchange constraints, gene expression data, the GIMME algorithm, and flux variability analysis. The two models detailed the metabolism of obese and type II diabetes obese individuals based on the prolonged starvation multi-tissue simulation. **(B) **We compared the reaction activity of the two context-specific models. First, we looked at how reactions were expressed based solely on the gene expression data (left column). Second, we looked at reaction activity by determining the flux variability of the two context-specific models from the expression data and GIMME algorithm (right column).

Context-specific multi-tissue networks were built using the GIMME algorithm [7] that maps transcription data onto the reconstruction removing the reactions associated with absent transcripts. Flux variability analysis is then used to determine and remove the reactions that cannot carry flux in both context-specific networks. The remaining reactions represent potentially active reactions under that context and provide a qualitative capacity/capability measurement of the network. In order to examine the differences between candidate metabolic functions of the two disease states, the reaction activity of both context-specific models were compared. The workflow is shown in Figure 7A.

There is a considerable difference in reaction activity between the two context-specific states (Figure 7B). 29 reactions are present only in the normal obese model while 94 reactions are present only in the diabetic obese model. The majority of the reaction activity difference is in the AM. In addition, the multi-tissue models were required for providing proper context for the gene expression data. Ignoring the reconstruction context, the gene expression data predicted 27 and 40 exclusively expressed reactions in the normal and diabetic obese patients, respectively. 39 of the 67 expression predicted reactions were not differentially active in the context-specific models. This difference was due to either the unexpressed reaction being added back in for growth rate requirements or that the expressed reaction was a false-positive in terms of the entire network. Thus, the context-specific models predicted 95 differentially expressed reactions due to stoichiometric and flux constraints that the expression data could not solely predict.

The reaction activity changes determined by the context-specific networks can be grouped into three main categories, as detailed below. The first two categories were differences that were not necessarily obvious, but understandable through known macroscopic physiological changes found in diabetic patients. Importantly, the multi-tissue model provides mechanistic explanations as to why the macroscopic physiological changes occurred. These two groups can be viewed as validations of the multi-tissue model and the importance of using a reconstruction, as almost all of the predictions made were not evident in the expression data. The third group consists of one reaction that was completely non-obvious and provides new and interesting metabolic insights into diabetes in obese individuals. A full listing of all differentially active reactions found in the expression data and the final context-specific models are provided in the Supplementary Material for further research (see Additional File 6).

The first group of differentially expressed reactions in the context-specific models deal with known metabolites that have elevated blood concentrations in diabetes: free fatty acids and lactate [26-28]. First, the AM diabetic obese model has many active fatty acid oxidation and carnitine shuttle reactions that are not active in the AM normal obese model. The hyperactivity in the diabetes model can be attributed to a diminished insulin response. The diminished insulin response during the fasting state leads to two attributes common in diabetics. There is a lack of regulation of lipolysis, beta-oxidation of triglycerides into free fatty acids, leading to elevated free fatty acid levels [29] and a lack of regulation of oxidation of fatty acids into acetyl-CoA. The increased breakdown of fatty acids into sugars partially accounts for observed hyperglycemia in diabetics as the fatty acids stimulate gluconeogenesis in the liver [30]. Inhibition of the fatty acid oxidation reactions that lead to the hyperglycemic effect has been previously proposed as a potential method to inhibit the condition [30].

Second, HM and MM lactate dehydrogenase are solely active in the normal obese patients. The absence of lactate dehydrogenase in diabetic patients is a potential metabolic mechanism for higher levels of lactate in the blood as the liver and muscle are unable to utilize lactate as a carbohydrate source. In addition, the differential activity of lactate dehydrogenase in the simulations demonstrates the power of the context of the multi-tissue simulation. The lactate dehydrogenase reactions were not differentially expressed in the transcription data, but the model provided proper context for predicting proper reaction activity. An individual model simulation would not yield the same results because the degradation of triacylglycerol from the AM and protein from the MM is critical for proper fasting state contextualization. The lack of lactate dehydrogenase can potentially attribute to the macroscopic observation of high levels of lactate in the blood of obese diabetics, as compared to normal obese individuals [28].

The second group of reactions provides clues into the metabolic mechanisms of oxidative tissue damage seen in diabetic patients [31]. There were changes in catalase reaction activity throughout the three cell-specific portions of the multi-tissue models. Catalase is a ubiquitous enzyme in aerobic organisms. The enzyme decomposes hydrogen peroxide into oxygen and water. Hydrogen peroxide is generated by free radical via superoxide dismutase. In our context-specific models, catalase was solely active in the AM of the diabetic obese model, but was solely active in the MM of the normal obese model. It has been previously shown that the inherited disorders of acatalasemia and hypocatalasaemia, which lead to defective catalase, increase the likelihood of type II diabetes [32]. The proposed mechanism involves pancreatic beta cells, which are susceptible to oxidative damage. Goth and Eaton proposed that defective catalase increases oxidative species, thus destroying pancreatic beta cells. To detect the defect, erythrocyte catalase was studied. However, no previous studies have been done with the activity of healthy catalase enzyme. Simulations show that catalase activity is not present in the skeletal muscle of diabetic obese patients during the fasting state. Absence of catalase in a cell would have a similar functional effect as expression of defective catalase. The absence of catalase activity probably attributes to oxidative tissue damage as well as may play a role in diabetes development.

Thirdly, AM cysteine dioxygenase (CDO) is only active in the normal obese model. CDO has been shown to be an important regulator in cysteine and sulfur metabolism in adipocytes and hepatocytes [33] but has not been shown to be related to diabetes. HM CDO has activity in both context-specific models because the hepatic version of the enzyme is post-translationally regulated [33]. CDO is very responsive to dietary changes to protein and sulfur amino acid intake in normal individuals. It is responsible for breaking down excess cysteine into other important metabolites, such as pyruvate and taurine. Very little research has been done on CDO in diabetics. Elevated levels of cysteine in tissues have been shown to be cytotoxic and could be another potential mechanism for the observation of tissue damage in diabetic patients. In addition, taurine has been implicated as an important metabolite in diabetes and supplements of taurine have been shown to reduce diabetic symptoms [34,35]. The lack of CDO reaction activity in the diabetic context-specific model is a potential reason for the diminished taurine concentration.

## Conclusion

Fine-grained studies of integrated human metabolic states have proved to be difficult due to complex intracellular and intercellular interactions. The recently available human genome-scale reconstruction, Recon 1 [5], is a biological knowledgebase for studying intracellular human metabolism that now enables such an undertaking. This paper presents the first effort to build a multi-tissue metabolic network that is global in the sense that all metabolic functions described on the human genome are taken into account. Thus, three cell-specific genome-scale metabolic networks were reconstructed. They were integrated using a multi-tissue modeling approach and the integrated model was used to study physiologically relevant cycles. High-throughput data was integrated in the context of the integrated metabolic network to study differences in obese and diabetic obese individuals. Several findings resulted from this study.

First, using Recon 1 we generated cell-specific reconstructions for three key tissues involved in diabetes and obesity: adipocytes, hepatocytes, and myocytes. The three cell-specific reconstructions were converted into mathematical models and put through stringent testing to validate tissue-specific physiological functions.

Second, intercellular metabolic interactions were then described by developing a multi tissue-type modeling paradigm that combines the cell-specific models through a blood compartment. The multi-tissue model is not simply a trivial sum of the cell-specific reconstructions. As an example, a bicarbonate buffering system was required to simulate the mathematical model.

Third, using the multi-tissue models, simulations for the Alanine and Cori cycles and the absorptive state are possible. These three physiologically relevant cycles are presented. When comparing the nutrient limited cycles to the individual models (e.g. Alanine and Cori cycles), the multi-tissue simulation approach showed a constrained solution space. In a nutrient rich environment (e.g. absorptive state), the HM and MM were constrained while the AM was not. The AM is not as metabolically independent as the HM and MM and thus gains potential phenotypes when integrated with the other metabolic reconstructions.

Fourth, utilizing the multi-tissue approach, the metabolic differences in obese and diabetic obese individuals were studied by incorporating gene expression data as a constraint on the metabolic networks. The approach was validated and provided potential mechanisms for known macroscopic physiological changes seen in diabetic patients such as increased blood metabolite concentrations and oxidative damage of tissues. In addition, cysteine dioxygenase was found to be differentially active and could be a potential factor in oxidative damage to tissues and lower concentrations of taurine in adipose and liver tissues. The differences in the two context-specific models were not obvious and required the multi-tissue modeling approach, as the differential activity of reactions could not be ascertained from the transcription data alone.

The first genome-scale metabolic network reconstructed was *H. influenzae*, representing the first sequenced prokaryote [36]. Other prokaryotic genome-scale metabolic networks have been reconstructed (e.g. *M. tuberculosis *[37,38], *H. pylori *[39], *S. aureus *[40]). The most notable prokaryotic reconstruction is that for *E. coli *with many successive expansions [41-43]. The next step was reconstructing a eukaryotic cell, with the introduction of the *S. cerevisiae *metabolic network [44]. With the need for understanding human metabolism for the health sciences, Recon 1 was introduced. Recon 1 is a comprehensive knowledge base for human cells allowing integration of high-throughput data to build cell type specific models. In this study, a multi-tissue type modeling approach is detailed that allows for an increased understanding of intercellular interactions. Integrating high-throughput data allows for the study of pathophysiological states. Multi-tissue simulations can provide a basis for designing isotopomer flux experiments and allow for mapping flux results onto the network. Utilizing the multi-tissue models for designing and analyzing flux experiments can increase the accuracy and quantitative utility of the multi-tissue approach, further expanding the usefulness and necessity of genome-scale metabolic networks for studying the health sciences.

## Methods

### Building a Tissue Specific Metabolic Model

Using the human genome sequence and annotation, biochemical, and physiological data available through online databases and published literature, three metabolic networks were reconstructed for the human hepatocyte, myocyte, and adipocyte. A workflow for this procedure is shown in Figure 2. We began with tailoring Recon 1. The gene index was updated from Build 35 to Build 36.2 (the current release at the time). The GeneID numbers in Recon 1 are not unique and were replaced by the unique RefSeq transcript IDs. All transporters and lumped reactions were redone with proper elemental and proton balancing. Tissue specificity information was obtained from the UniProt (Universal Protein Resource) database [45]. An automated draft model was reconstructed in SimPheny from the cell-specific open reading frames determined from UniProt and the updated Recon 1 model.

The draft models were finalized through manual curation. Metabolic pathways in each cell type were included based on the existing knowledge of cell physiology and cell-specific biomass requirements. For each pathway, the presence of each reaction was supported by one or more of the following information obtained from online databases and/or published literature: biochemical data, genetic data, localization data, sequence data, physiological data, and modeling data. Reaction properties were verified through online databases such as KEGG [46] (for stoichiometry and cofactor specificity), NCBI (for organism specificity), UniProt (for localization), and BRENDA [47] (for reversibility, localization, and tissue-specificity). Presence, mechanism, and localization of metabolic pathways were also verified using textbook references [48-51] and tissue specific gene expression data (cDNA library, NCBI). If possible, reactions with dead-end substrates and products were deleted from, or interconnected within, the network to reduce metabolic gaps. Pathways for synthesis of known essential cell components, including the essential amino acids, vitamins, and fatty acids, were removed if present, and appropriate transport reactions were included to allow the uptake of the essential cell components into the network. Additional metabolic pathways that were not included in Recon 1 but were present in the tissue specific models were also added at this stage. For example, ketogenesis is present in the HM but not in Recon 1 and was accordingly added to the reconstruction. To preserve a standardized QC/QA procedure for building the three networks, the HM was not augmented with previously published liver metabolic reconstructions [8,9]. The additional hepatic metabolism covered in these two models is outside of the scope of multi-tissue interactions studied. A comparison of the two previous models and the one in this study is presented in the Supplementary Material (Additional File 7).

### Biomass Formulation

Biomass maintenance functions were constructed for the three cell-specific metabolic models. The process involved gathering primary literature data on the metabolic make up of the human tissue or related mammals if human-specific data was scarce. The overall process of formulating biomass objective functions for eukaryotes has been described in previous publications [44]. The composition of the HM is broken down into proteins, neutral lipids, phospholipids, glycogen, DNA, and RNA. The MM biomass function consists of non-collagen protein, collagen protein, lipids, DNA, RNA, and glycogen. The AM is composed of carbohydrates protein, lipids, DNA, and RNA (Figure 1C). A full breakdown of each macromolecular component and final weighting in the objective function is provided in the Supplementary Material (see Additional File 1).

### Integrating Metabolic Models

The overall compartment schematic of the integrated multi-tissue model is presented in Figure 4. There were two main steps for integration: mathematical integration and blood compartment refinement. For the first step, the reactions and metabolites in the three cell-specific models were renamed for proper compartmentalization. A new blood compartment representing the interstitial fluid, urine, and blood was constructed. The exchanges of the cell-specific models were removed and only gene-associated transporters and free diffusion allow for intercellular metabolite transport. Exchange reactions were added to allow the blood compartment to uptake and secrete metabolites into the extra-system. The second step involved refining the blood compartment. It was not possible to properly simulate using flux balance analysis due to improper proton balancing. A bicarbonate buffer reaction, similar to the function of the kidney, was added to account for proton balancing.

Other metabolic changes and degradation in the blood were assumed to be negligible due to the large time-scale differences between small metabolite stability in whole blood versus the amount of time a metabolite spends in the blood for transport. In particular, Liu et al. have shown through time course metabolomic profiling of plasma drawn from incubated whole blood that most small metabolites accounted for in the multi-tissue model's blood compartment are stable (25 of 33 metabolites showed no significant change in the 4 hr study) [52]. The rest of the metabolites are stable until the later time points, except for pyruvate that showed significant change by the first time point (1 hr). All originally detected metabolites were still present at the end of the study. On the other hand, blood circulates throughout the body in about a minute. The microvasculature is structured in a way for convective forces to dominate diffusive forces to increase the rate of uptake of solutes. In particular, metabolic uptake is in the time scale of seconds and minutes [53]. Such a large time scale difference (two to four orders of magnitude) makes blood degradative processes negligible. The metabolites spend only a few minutes in the blood compartment while their degradation takes hours. Thus, the amount of degradation in the blood compartment is negligible as metabolites are primarily located in the cells and tissues and it can be assumed that metabolic changes occur predominantly there.

In the human body, the three tissues have varying masses. Initially, the simulations were set up using the units: mmol/h/g cell DW; which assumes that all three tissues have the same weight. To properly simulate intercellular fluxes, the biomass objective functions were scaled to the units: mmol/h/body. Thus, the three tissue model fluxes were scaled to represent the entire tissue mass in the average human body. The units were scaled by the mass of the tissue [54-56], the cellular composition of the tissue, and subtracting the water weight (see Additional File 1). For the white adipose tissue and skeletal muscle tissue, it was assumed that adipocyte and myocyte were the cells present. For the HM, it was assumed that 80% of the cellular mass of the liver are hepatocytes [57].

### Flux Balance Analysis

After reconstruction in the SimPheny platform, the cell-specific metabolic models were converted into a mathematical format for analysis. The reactions and metabolites were represented with the stoichiometric matrix (**S**). The stoichiometric matrix is very sparse and has dimensions of **m **× **n**. The metabolites (**m**) are represented in the rows as nodes in the network, while the reactions (**n**) are represented in the columns as links. Ordinary differential equations for the time derivatives of the metabolic concentrations are set up for the reconstruction using the stoichiometric matrix and reaction fluxes (**v**):

(1) $$\frac{dx}{dt}=S\cdot v$$

Due to the lack of kinetic parameters available for intracellular biochemical transformations at the genome-scale, Equation 1 is set to a steady state and flux balance analysis (FBA) is used to characterize the system. FBA uses linear programming techniques to calculate the flow of metabolites throughout the network under mass balance (**S·v **= 0) and thermodynamic constraints (**lb**, **ub**) (Equation 2). For those unfamiliar with linear programming and FBA, a primer is now available [58].

(2) $$\begin{array}{c} \max\left(c^{T}\cdot v\right)\\ subject\;to\;S\cdot v=0\\ lb\;<\;v\;<\;ub \end{array}$$

Characterizing the extremities of the system's solution space is done using a variant of FBA called flux variability analysis (FVA). FVA involves iteratively and independently determining the minimum and maximum fluxes through the metabolic network. The flux span for a reaction is defined as the difference between the maximum and minimum flux. When analyzing the flux span, we did not consider reactions that were perceived to be part of thermodynamically infeasible internal loops.

### Context-Specific Multi-tissue Simulation

Context-specific metabolic networks for obese and diabetic obese individuals were built using the prolonged starvation multi-tissue model and the Gene Inactivity Moderated by Metabolism and Expression (GIMME) algorithm [7]. The GIMME algorithm is a linear programming problem that uses gene expression data to minimize the flux of down regulated genes. The procedure results in a smaller context-specific model built from the original multi-tissue model as unexpressed reactions are removed from the metabolic network. Gene expression data for obese and diabetic obese individuals was obtained through the Gene Expression Omnibus (GSE15773, GSE15653, GSE18732) [59,60]. Expression data was normalized using GCRMA and presence and absence calls were made using the PANP function in the R statistical platform (p < 0.01) for each patient group (diabetic, non-diabetic) of each tissue (adipose, liver, muscle). For the GIMME simulations, genes were deemed present if they were present within all samples of the particular tissue and group. Before using GIMME, a permutation sensitivity analysis was completed by removing up to 50% of the samples from each tissue and patient group. The expression data was very stable for all groups (see Additional File 8). Flux variability analysis was used to compare which reactions could carry flux in both context-specific models and thus ascertain the metabolic differences in reaction activity between obese and diabetic obese individuals.

## Authors' contributions

AB updated and integrated the networks, performed the analyses, and drafted the manuscript. RU, JW, and IF constructed the initial networks. AMF, BOP, IF conceived the study and revised the manuscript. All authors approved the content of the final manuscript.

## Supplementary Material

Additional file 1 **Biomass Formulation**. Supplementary information on sources and methods used to constract tissue-specific biomass functions. Click here for file

Additional file 2 **Tissue specific models and multi-tissue model XLS (in compressed zip format)**. Metabolic models for adipocyte, hepatocyte, mycocyte, and multi-tissue network in XLS format Click here for file

Additional file 3 **Tissue specific models and multi-tissue model SBML (in compressed zip format)**. Metabolic models for adipocyte, hepatocyte, myocyte, and multi-tissue network in SBML format. The SBML format can be imported into Matlab and COBRA Toolbox for simulations Click here for file

Additional file 4 **QC/QA Tests and Futile Cycle Tests**. Quality control tests to validate universal and tissue specific functionality and model consistency Click here for file

Additional file 5 **Unconstrained simulations of Cori and Alanine Cycles**. Additional information on simulating the metabolic interaction between the liver and muscle based on sensitivity analysis of objectives (hepatic glucose and urea production) Click here for file

Additional file 6 **Reaction Activity Changes in Obese and Diabetic Obese Individuals**. Differential metabolic reactions in simulations of obese and diabetic obese individuals determined by flux variability analysis Click here for file

Additional file 7 **Liver metabolic network reconstruction comparison**. Comparison of HM, Recon 1, and two existing liver metabolic networks in terms of metabolites and reactions. Click here for file

Additional file 8 **Expression Data Sensitivity Analysis**. Sensitivity analysis involving removing random permutations of patient samples from each group to determine robustness of expression data. Click here for file
