# Supplementary material for: A multi-tissue type genome-scale metabolic network for analysis of whole-body systems physiology
Source: BMC Syst Biol. 2011 Oct 31;5:180. doi: 10.1186/1752-0509-5-180 (PMC3219569; doi:10.1186/1752-0509-5-180)
Supplement: Additional file 1 — Biomass Formulation. Supplementary information on sources and methods used to constract tissue-specific biomass functions. [file 1752-0509-5-180-S1.DOC]

### Hepatocyte Biomass Composition

In the absence of direct experimental measurements for the human liver, published data for rat was used to define the protein, lipid, carbohydrate, and nucleotide content in the liver tissue (Table 1) (Davidson, J. N., 1957;Bar, A*. et a*l., 1999).

**Table 1.** Composition of the rat liver cell. The average values were generated using the male and female cell composition. Adopted from (Davidson, J. N., 1957).


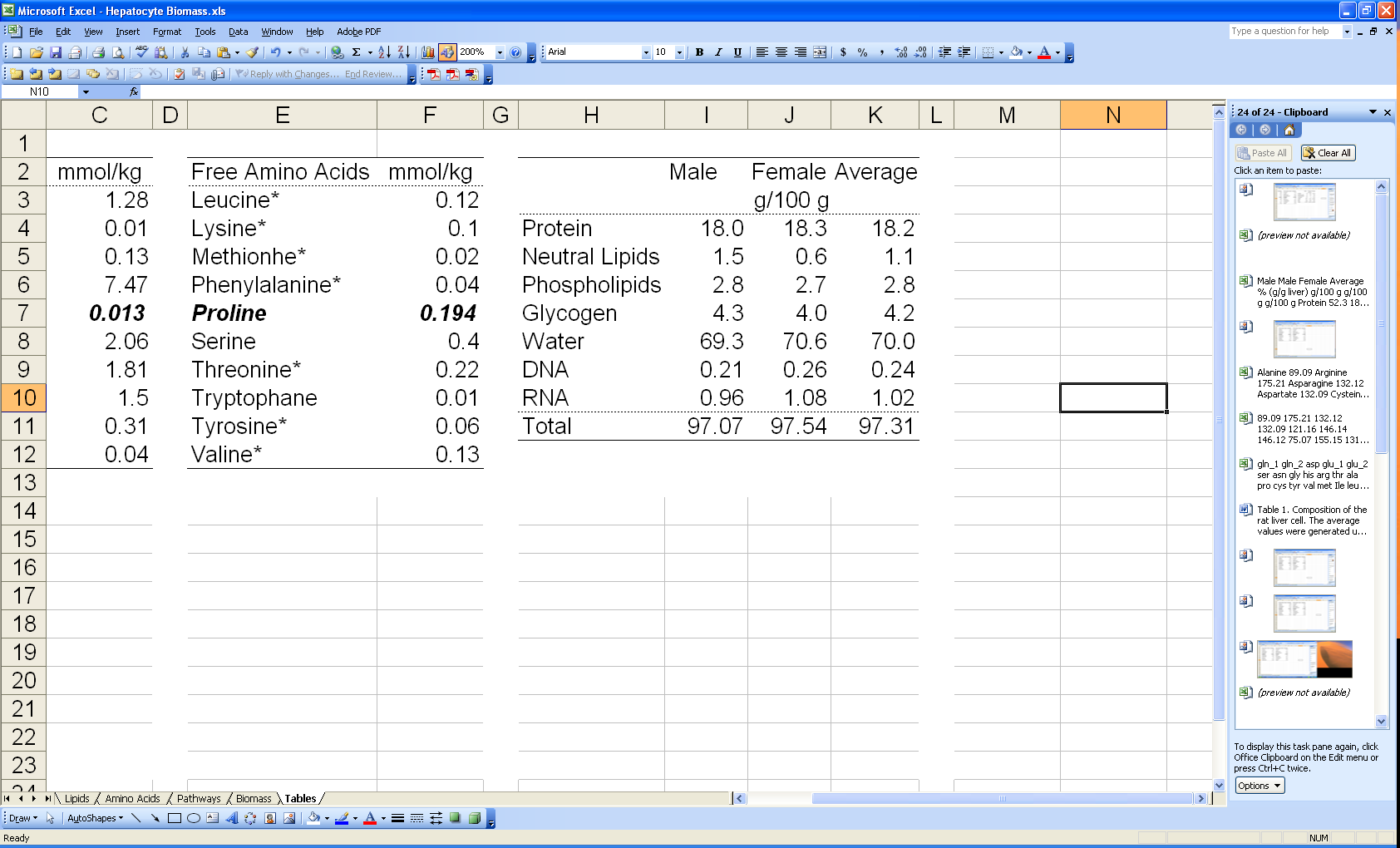


Since no reference for the total amino acid composition of the liver was found, the free amino acid composition of the human liver was used to represent the total amino acid content of the liver tissue (Table 2) (Barle, H*. et a*l., 1996). Since the free amino acids in the rat liver tissue were similar to that of the human liver, reported values for cystein and proline in rat were used for the missing amino acids in the human liver data (Triguero, A*. et a*l., 1997). Note that the free amino acid composition is most likely different from the total amino acid composition in the cell; however, since the amino acid content and biomass formation rate constitute a very small portion of the demands imposed on the metabolic network during the flux balance simulations, the associated error is not of major concern at this point.

**Table 2.** Free amino acid composition of the human and rat liver cell. All values correspond to the human data (Barle, H*. et a*l., 1996), except for the bold values adopted from rat liver data (Triguero, A*. et a*l., 1997).


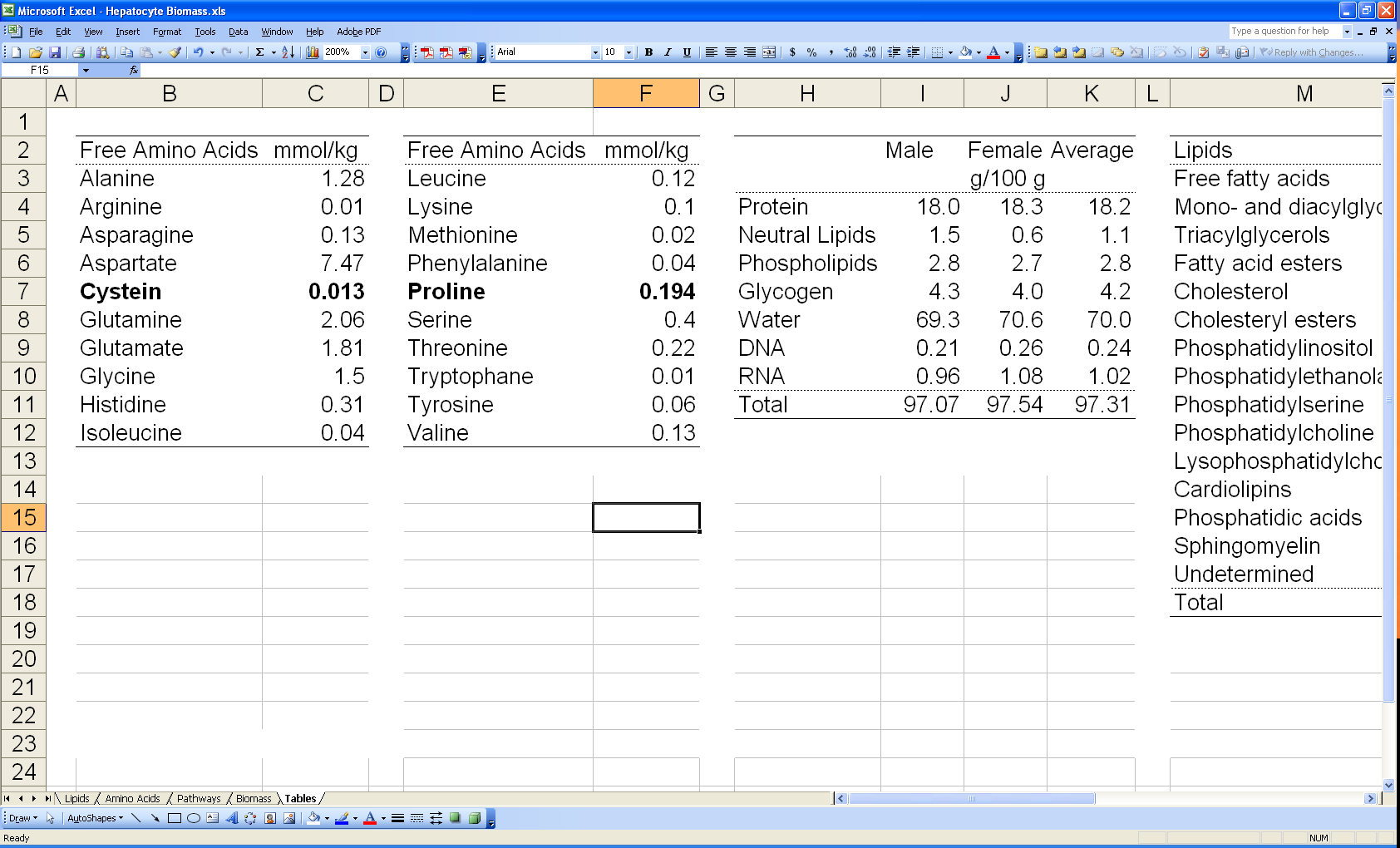


The lipid distribution of the human liver has been measured for the neutral lipid and phospholipids (Table 3) (Rabinowitz, J. L*. et a*l., 1992).

**Table 3.** Lipid composition of the human liver tissue. Adopted from (Rabinowitz, J. L*. et a*l., 1992).


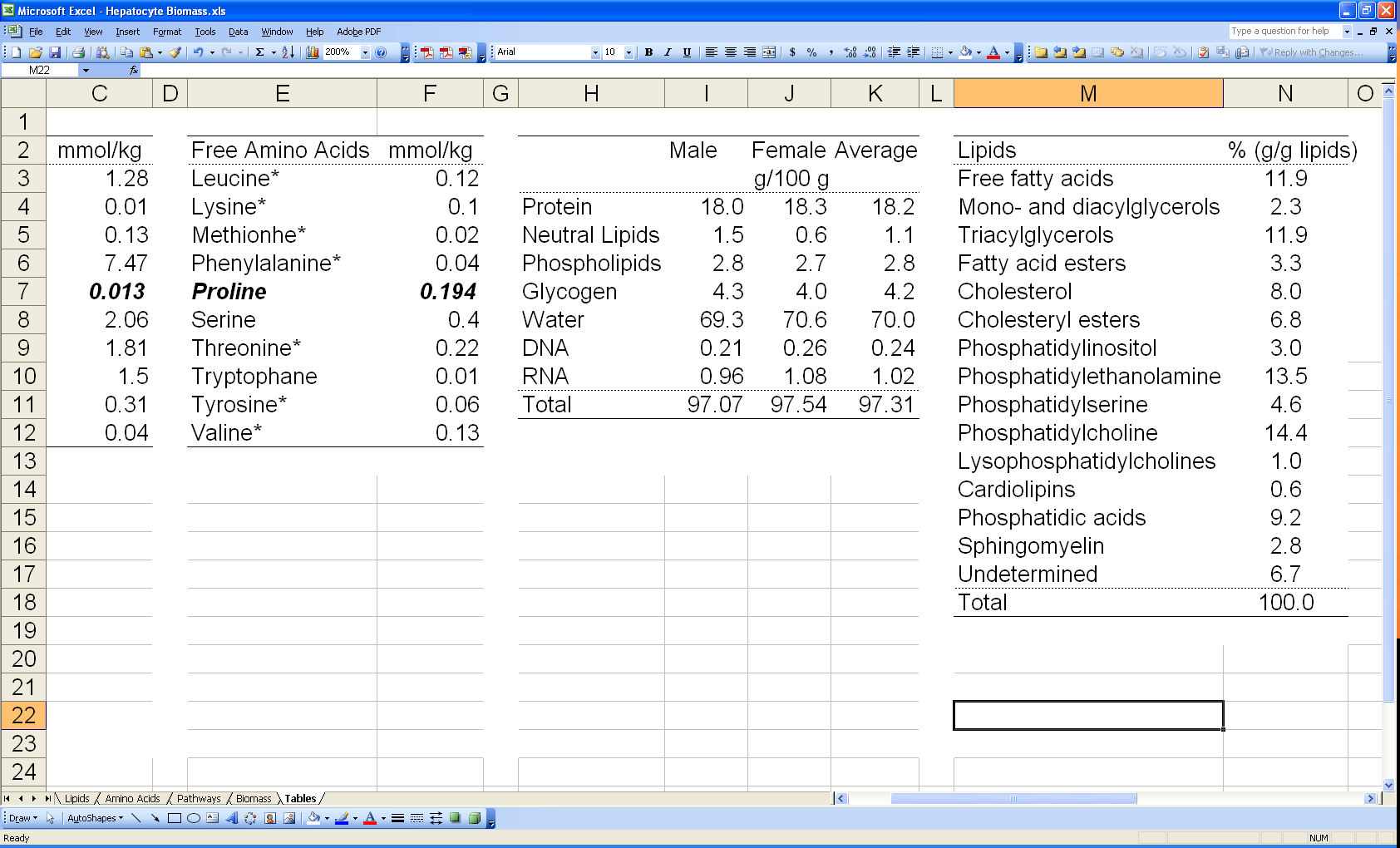


Fatty acid composition of triglyceride in normal human liver sample tissue has been measured and reported previously (Kang, E. S*. et a*l., 1991). Normalized values (i.e. rounded up to 100%) of the fatty acid components were used to define the molecular formula and balanced metabolic reactions in triglyceride and phospholipid metabolism in the hepatocyte network and to identify the additional reactions and pathways that were required to be added to the reconstructed network (Table 4).

**Table 4.** Fatty acid composition of triglycerides extracted from control human liver samples. Adopted from (Kang, E. S*. et a*l., 1991).


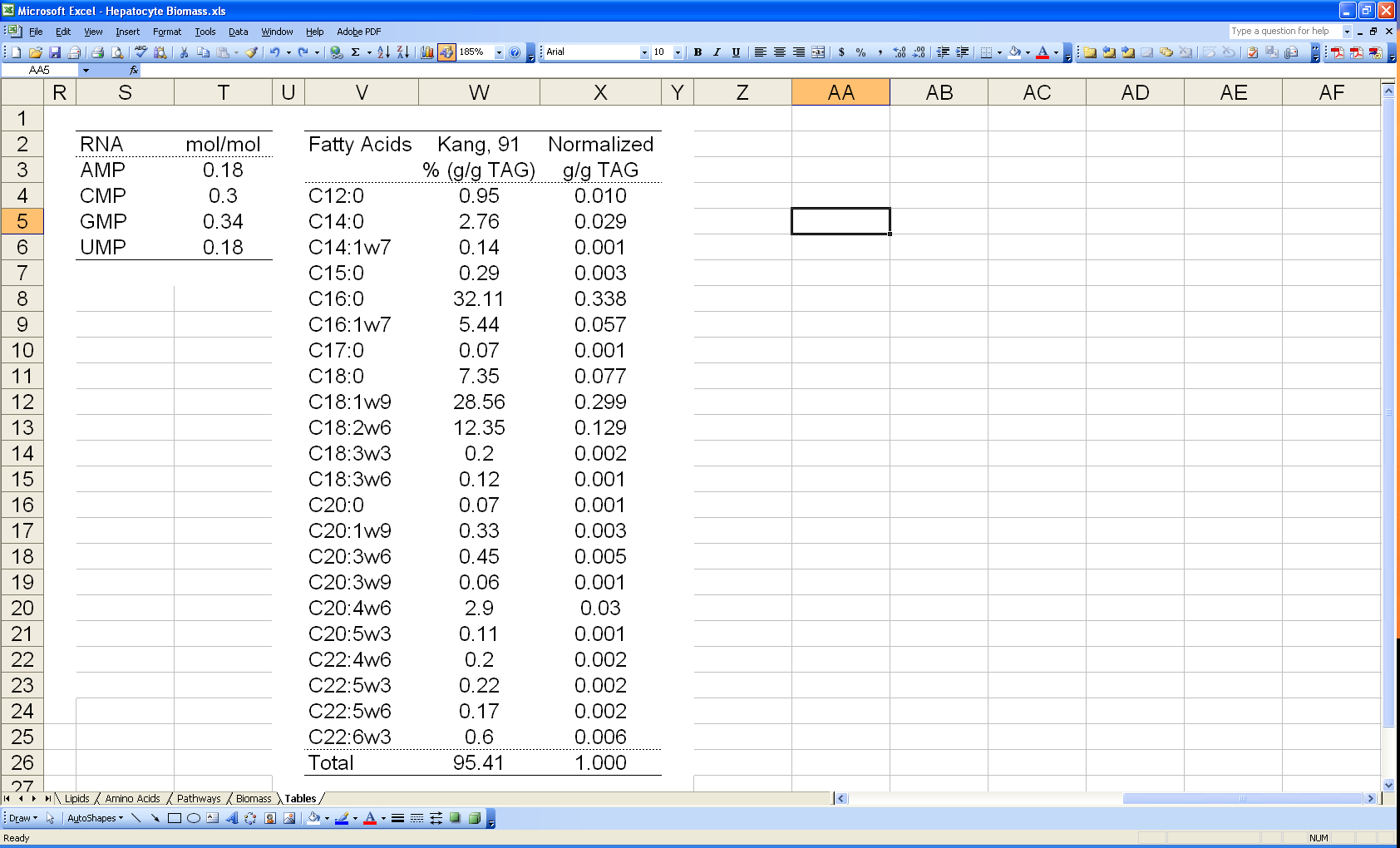


The nucleotide composition of the liver from gene expression studies (Teufel, A*. et a*l., 2007) have shown to be similar to those reported previously for animal cells (Sheikh, K*. et a*l., 2005). Thus, in the absence of available measurements for the human liver tissue, values reported for animal cells were used for nucleotide composition of the hepatocyte network (Table 5) (Sheikh, K*. et a*l., 2005).

**Table 5.** Nucleotide composition of the animal cell. Adopted from (Sheikh, K*. et a*l., 2005).


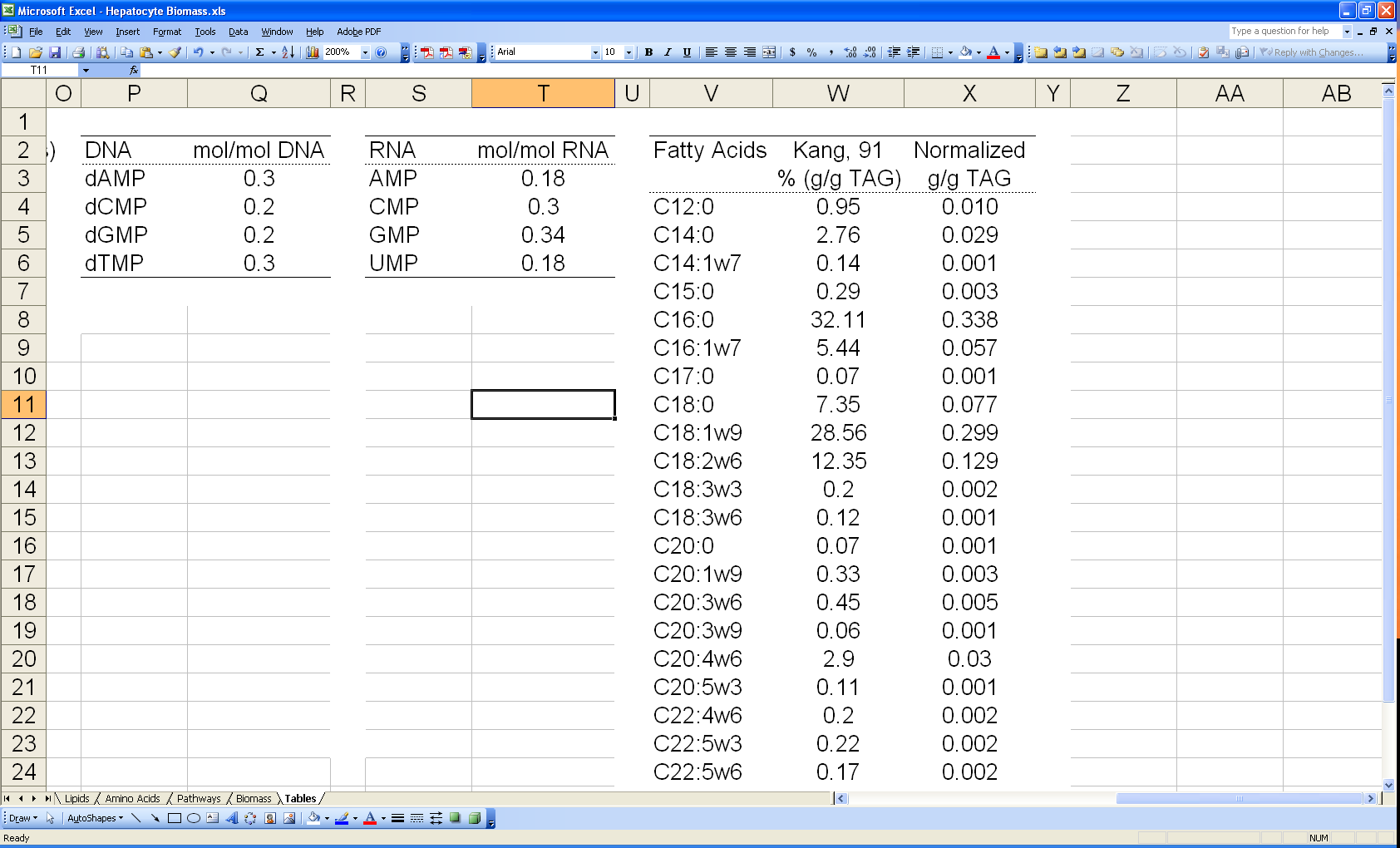


### Myocyte Biomass Composition

Biochemical composition of human muscle has been determined previously (Heymsfield, S. B*. et a*l., 1982) and used as myocyte biomass composition in this study (Table 6).

**Table 6.** Composition of the human muscle. Adopted from (Heymsfield, S. B*. et a*l., 1982).

The free amino acid composition (Pouw, E. M*. et a*l., 1998) and protein-bound amino acid composition (Mackenzie, B*. et a*l., 1993) from human muscle were used to represent the total amino acid content of the muscle tissue (Table 7).

**Table 7.** Free amino acid composition and protein-bound amino acid composition of human skeletal muscle. Free amino acid values adopted from (Pouw, E. M*. et a*l., 1998). Protein-bound amino acid data adopted from (Mackenzie, B. et al., 1993). Amino acids marked with a star indicate essential amino acids. Amino acid concentrations are expressed in mmol per kg of wet weight of muscle tissue. * indicate essential amino acids.

The lipid distribution of the human skeletal muscle has been measured for the total lipid (Table 8) (Aas, V*. et a*l., 2004;Frattola, L*. et a*l., 1974) and phospholipids specifically (Table 9) (Bruce, A., 1974). This data was adopted for myocyte biomass calculation. Fatty acid composition of human skeletal muscle phospholipid and triglyceride has been measured and reported previously (Andersson, A*. et a*l., 2000). For primarily network reconstruction normalized values (i.e. rounded up to 100%) of the fatty acid components of phospholipids were used to define the molecular formula and balanced metabolic reactions in triglyceride and phospholipid metabolism in the myocyte network (Table 9). The fatty acid composition of the human skeletal muscle phospholipid (Table 10) (Andersson, A*. et a*l., 2000) was used to identify reactions and pathways in the human muscle lipid metabolism. These identified reactions and pathways of lipid metabolism were added to the reconstructed myocyte network.

**Table 8.** Total lipid composition of the human skeletal muscle tissue. Adopted from (Frattola, L*. et a*l., 1974).

**Table 9.** Phospholipid composition of the human skeletal muscle tissue. In the reference, lipid concentrations were expressed using % of total lipid phosphorus. This corresponds to mol lipid per mol of total lipid. Adopted from (Bruce, A., 1974).

**Table 10.** Fatty acid composition of phospholipids extracted from healthy human skeletal muscle samples. Fatty acid concentrations are expressed in gram-% per gram of wet weight of muscle tissue. Adopted from (Andersson, A*. et a*l., 2000).

Previous studies show that nucleotide composition among various mammal tissues is compatible (Sheikh, K*. et a*l., 2005;Teufel, A*. et a*l., 2007;Kopteva, L. A*. et a*l., 1966). The nucleotide composition of the dog heart muscle (Kopteva, L. A*. et a*l., 1966) has been examined spectrophotometrically and adopted in this study for biomass calculation in human myocyte model (Table 11).

**Table 11.** Nucleotide composition of the mammal heart muscle used in the human myocyte network. Mononucleotide concentrations are expressed in mol % per mol of nucleic acid. Adopted from (Kopteva, L. A*. et a*l., 1966).

### Adipocyte Biomass Composition

Macromolecular composition of the adipose tissue was determined by collecting data from published measurements of the human and rat adipose tissue (Braun *T et a*l., 1968;THOMAS, L. W., 1962;Baker, G. L., 1969). With the exception of the total carbohydrate content, the protein, lipids, nucleotides, and water content was taken from measured values in the human adipose biopsies or samples (Table 12). Although a slightly different lipid composition of 68% has been reported for the human adipose tissue by Thomas (THOMAS, L. W., 1962), the values reported by Baker (Baker, G. L., 1969) were used since it is consistent with other reported values of the water and lipid content in the mammalian adipose tissue (Bernard, A*. et a*l., 1988).

**Table 12.** Composition of the adipose tissue.


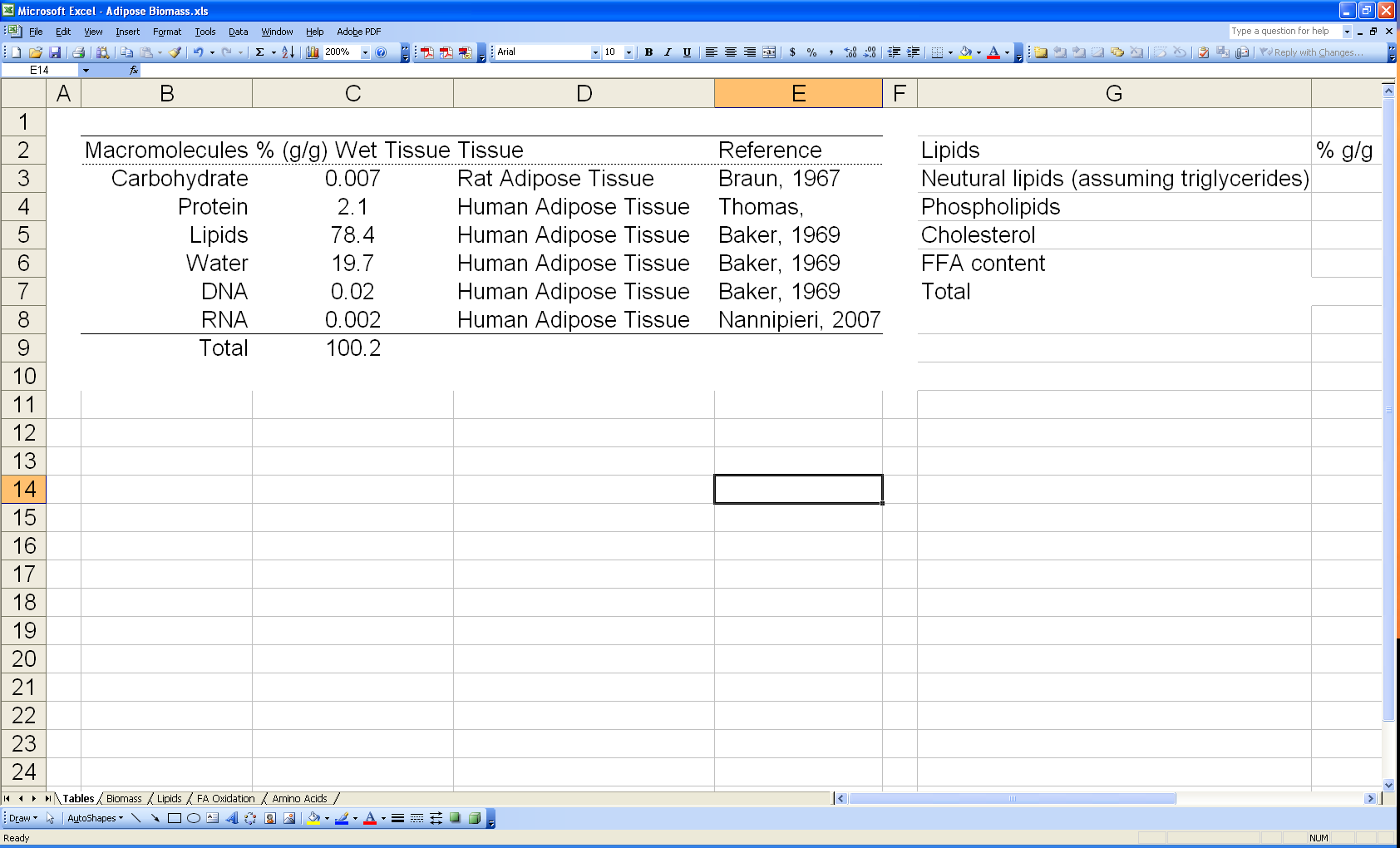


The amino acid composition was adopted from the measured values in the rat epididymal adipose tissue (CHRISTOPHE, J*. et a*l., 1963).

**Table 13.** Free amino acid composition of the rat epididymal adipose tissue. Adopted from (CHRISTOPHE, J*. et a*l., 1963).


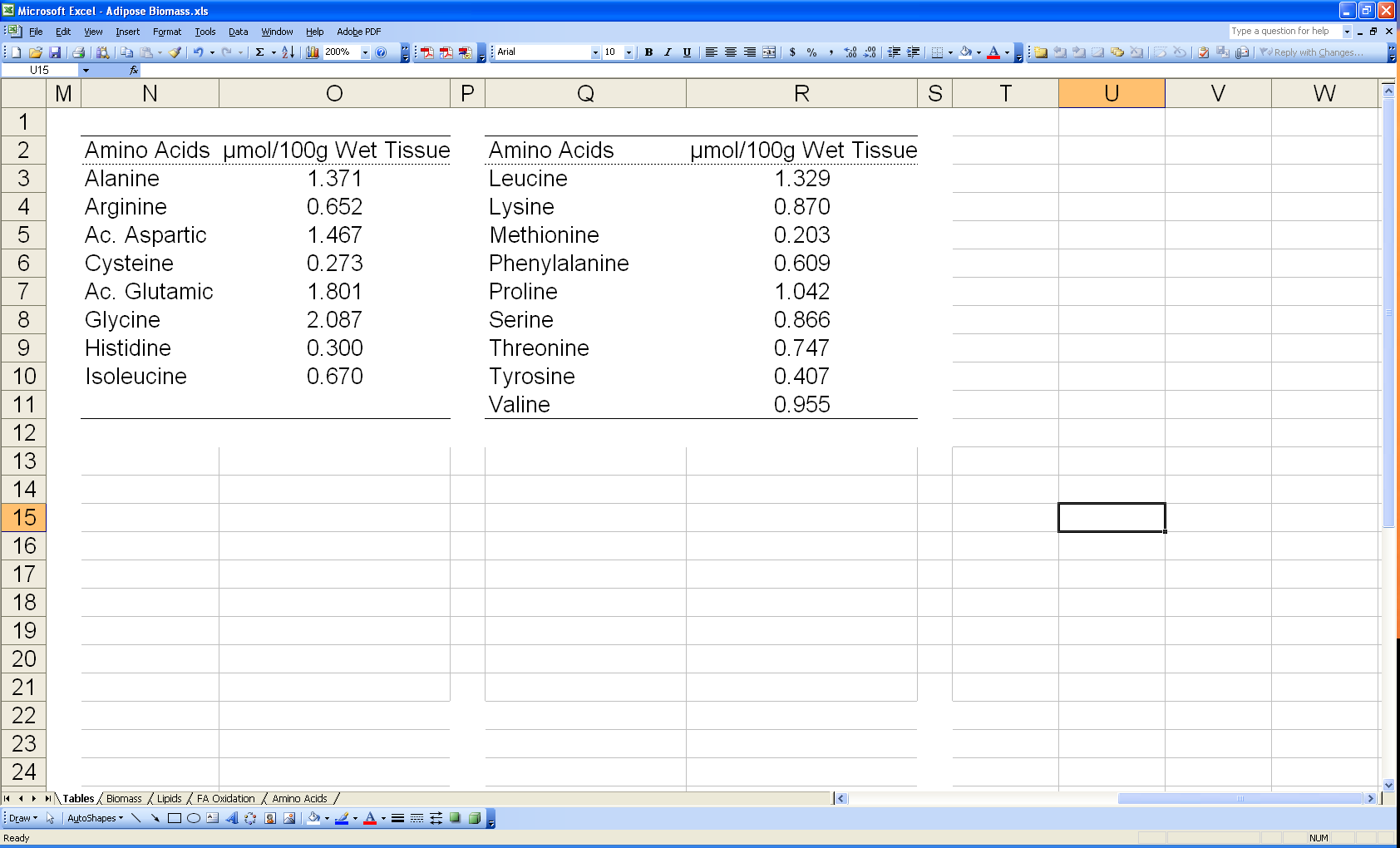


The lipid content of the normal human adipose subcutaneous tissue has been measured and reported previously (Table 14) (Banerjee, A. K*. et a*l., 1983). Neutral lipids were assumed to be entirely composed of triglycerides.

**Table 14.** Lipid composition of the normal human subcutaneous adipose tissue. Values are reported in percent weight of the wet adipose tissue. Adopted from (Banerjee, A. K*. et a*l., 1983).


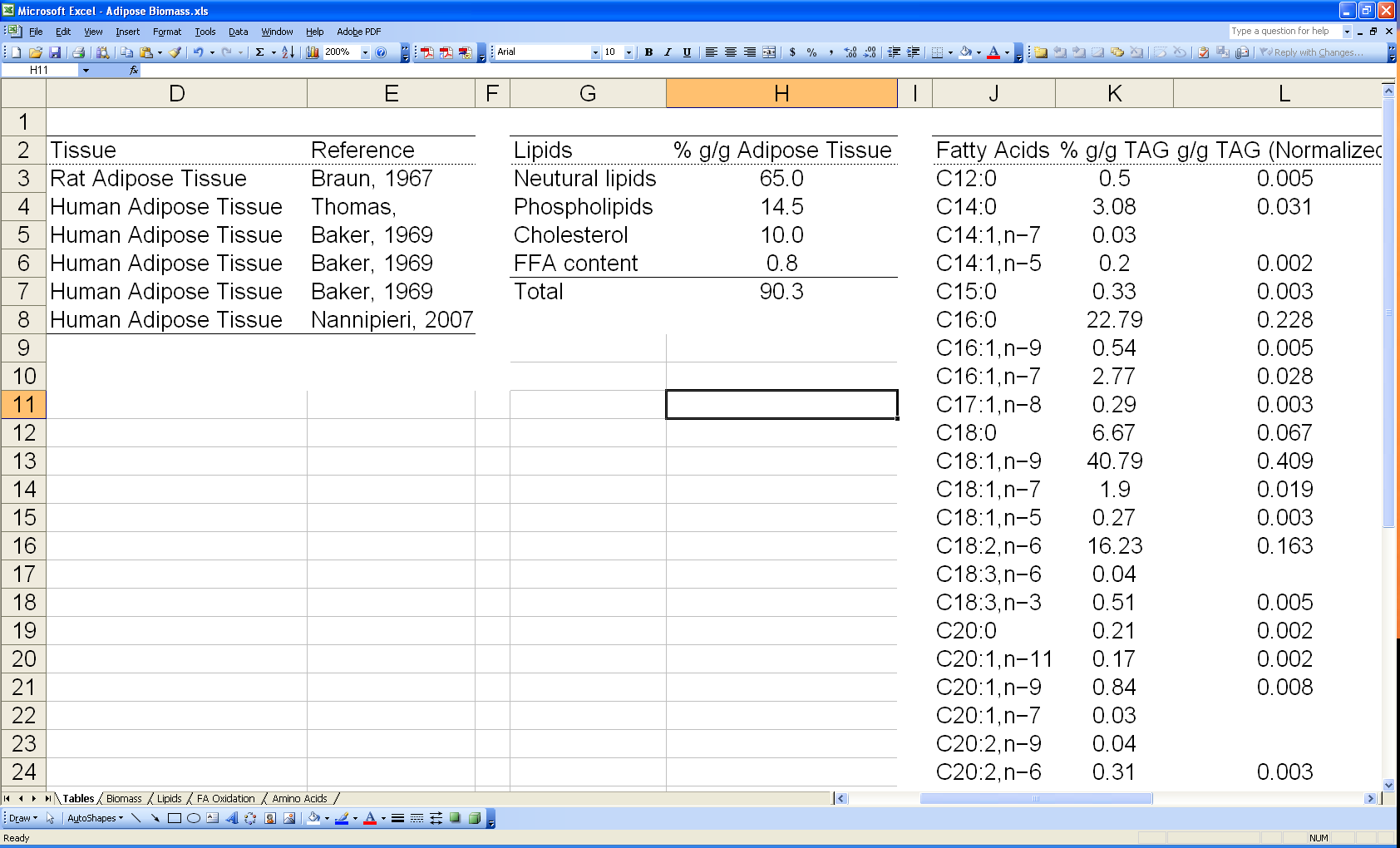


Total phospholipid composition of the adipose tissue has also been reported for the normal human subcutaneous adipose tissue (Table 15) (Banerjee, A. K*. et a*l., 1983).

**Table 15.** Phospholipid composition of the normal human subcutaneous adipose tissue. Adopted from (Banerjee, A. K*. et a*l., 1983). PC, Phosphatidylcholine; PE, phosphotidylethanolamine; PI, Phosphatidylinositol; PS, Phosphatidylserine


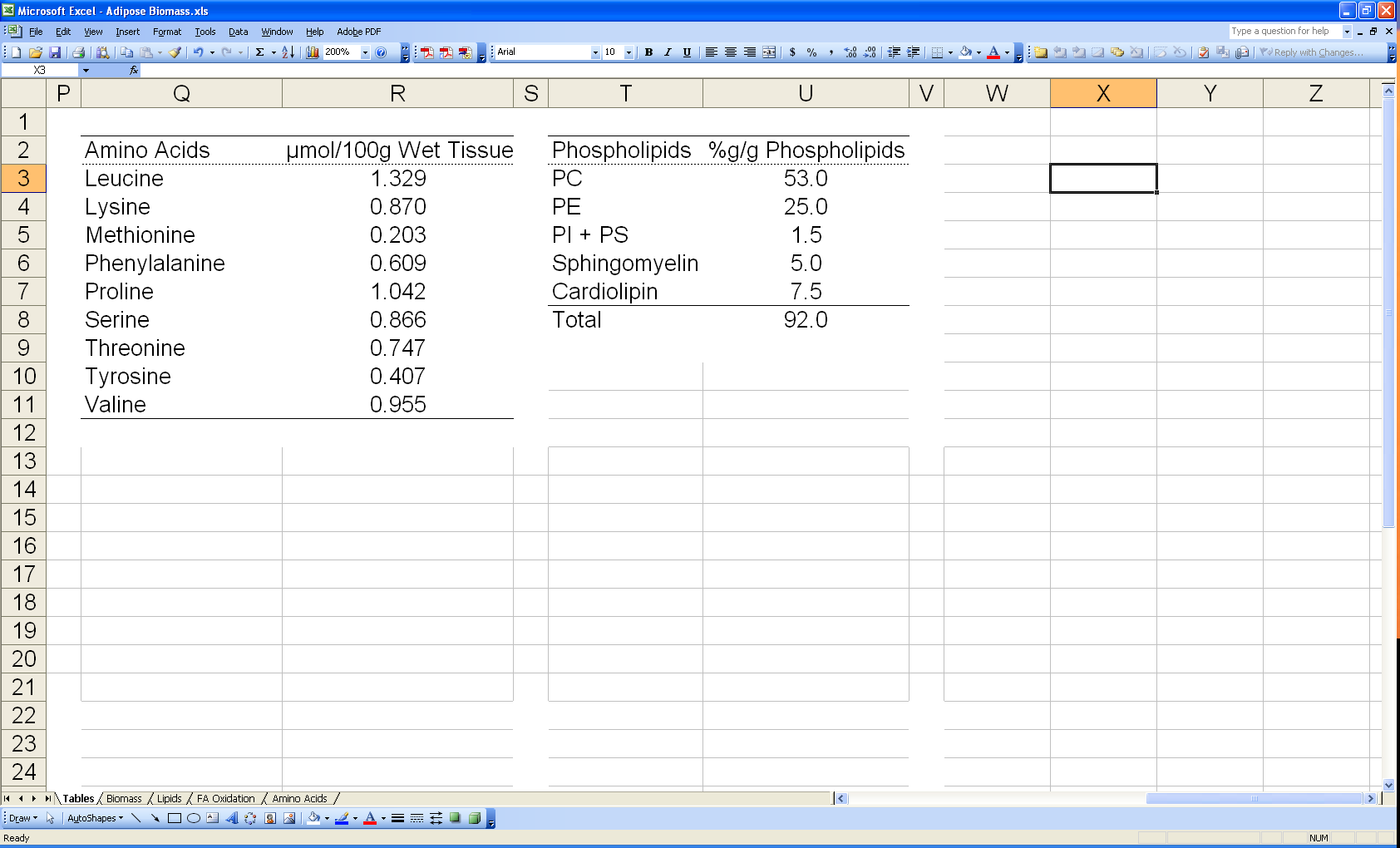


Fatty acid composition of the adipose tissue has been measured in a number of studies. Fatty acids from the normal breast adipose tissue were used in the adipocyte network to determine the fatty acid biosynthetic pathways and to define the molecular formula and balanced metabolic reactions in triglyceride and phospholipids (Table 16) (Raclot, T*. et a*l., 1997). The reported fatty acid content was normalized to 1 g/g of triglyceride (TAG) and fatty acids with values of smaller than 0.0005 g/g TAG were ignored for simplification.

**Table 15.** Fatty acid composition of triglycerides extracted from control human breast adipose tissue. Fatty acids with normalized values of smaller than 0.0005 g/g of triglyceride (TAG) were ignored for simplification. Adopted from (Raclot, T*. et a*l., 1997).


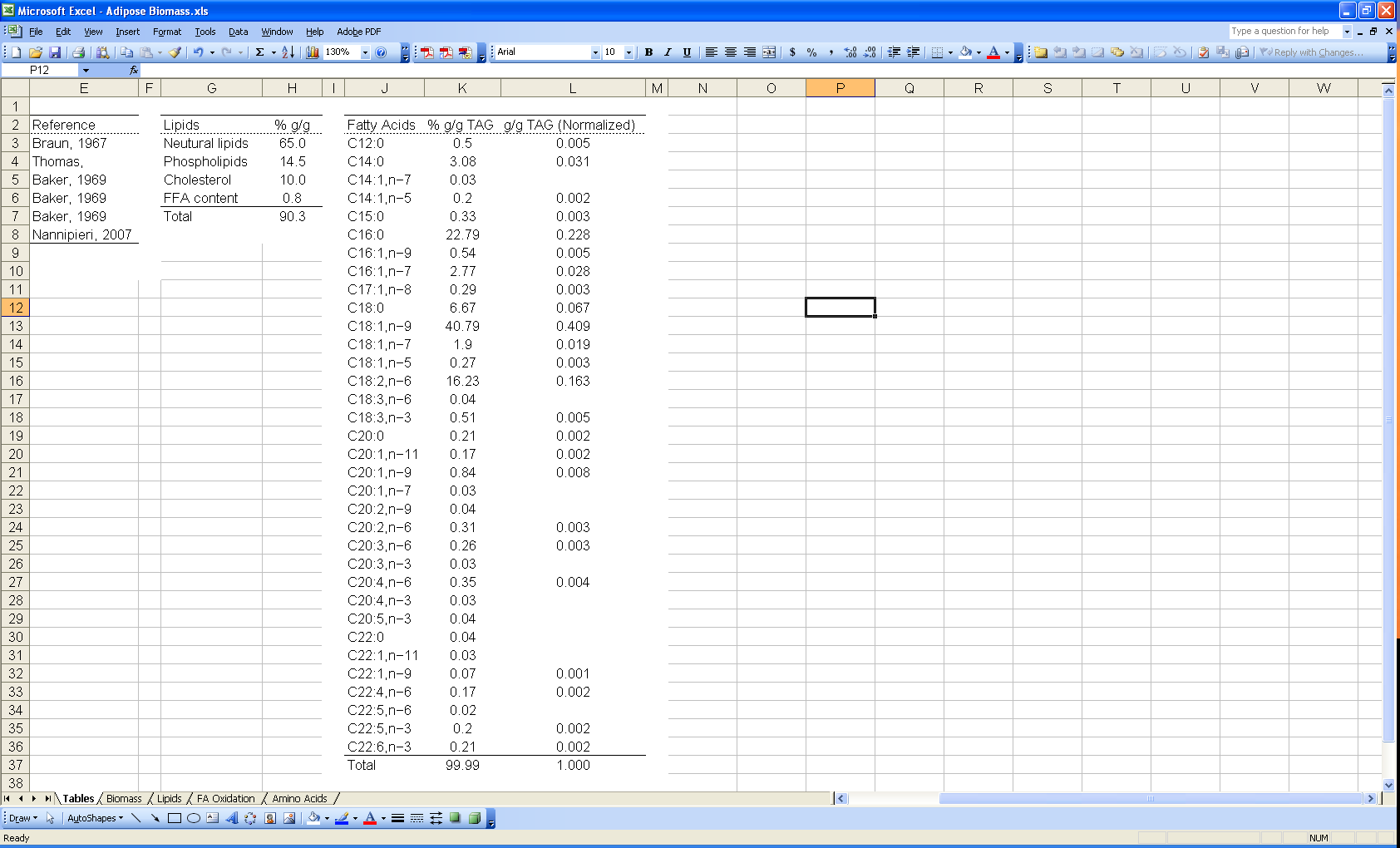


The nucleotide composition of the adipocyte was taken from the previously reported values for animal cells, as described previously (Table 5) (Sheikh, K*. et a*l., 2005).

**References**

Aas, V., Kase, E. T., Solberg, R., Jensen, J., and Rustan, A. C. (2004). Chronic hyperglycaemia promotes lipogenesis and triacylglycerol accumulation in human skeletal muscle cells. Diabetologia **47:** 1452-1461.

Andersson, A., Sjodin, A., Hedman, A., Olsson, R., and Vessby, B. (2000). Fatty acid profile of skeletal muscle phospholipids in trained and untrained young men. American Journal of Physiology-Endocrinology and Metabolism **279:** E744-E751.

Baker, G. L. (1969). Human adipose tissue composition and age. Am.J Clin.Nutr. **22:** 829-835.

Banerjee, A. K. and Goyle, S. (1983). Altered lipid composition of adipose tissue in human muscular dystrophy. Biochem.Med **30:** 246-252.

Bar, A., Lina, B. A., de Groot, D. M., de Bie, B., and Appel, M. J. (1999). Effect of D-tagatose on liver weight and glycogen content of rats. Regul.Toxicol.Pharmacol. **29:** S11-S28.

Barle, H., Ahlman, B., Nyberg, B., Andersson, K., Essen, P., and Wernerman, J. (1996). The concentrations of free amino acids in human liver tissue obtained during laparoscopic surgery. Clin.Physiol **16:** 217-227.

Bernard, A., Zwingelstein, G., Meister, R., and Wild, T. F. (1988). Hyperinsulinemia induced by canine distemper virus infection of mice and its correlation with the appearance of obesity. Comp Biochem.Physiol B **91:** 691-696.

Braun T, Vrana A, and Fabry P (1968). Enhanced Hypoglycaemic Effect of Exogenous Insulin Associated with an Increased Response of Adipose Tissue and a Diminished Response of Diaphragm in Meal Fed Rats. Experientia **23:** 468-470.

Bruce, A. (1974). Skeletal-Muscle Lipids .2. Changes in Phospholipid Composition in Man from Fetal to Middle-Age. Journal of Lipid Research **15:** 103-108.

CHRISTOPHE, J. and WODON, C. (1963). [AMINO ACID COMPOSITION OF THE EPIDIDYMAL ADIPOSE TISSUE OF THE NORMAL RAT.]. Arch.Int.Physiol Biochim. **71:** 720-740.

Davidson, J. N. (1957). Chemistry of the Liver Cell. British Medical Bulletin **13:** 77-81.

Duarte, N. C., Becker, S. A., Jamshidi, N., Thiele, I., Mo, M. L., Vo, T. D., Srivas, R., and Palsson, B. O. (2-6-2007). Global reconstruction of the human metabolic network based on genomic and bibliomic data. Proc Natl Acad Sci U S.A **104:** 1777-1782.

Frattola, L., Canal, N., Andreoli, V. M., Maffei, F., and Tonon, G. C. (1974). Lipid Changes in Neurogenic Muscular Atrophies. Research in Experimental Medicine **164:** 241-246.

Guyton, A. C. (1991). "Textbook of Medical Physiology", Saunders.

Heymsfield, S. B., Stevens, V., Noel, R., Mcmanus, C., Smith, J., and Nixon, D. (1982). Biochemical-Composition of Muscle in Normal and Semi-Starved Human-Subjects - Relevance to Anthropometric Measurements. American Journal of Clinical Nutrition **36:** 131-142.

Kang, E. S., Johnson, S. B., and Holman, R. T. (12-31-1991). Fatty acid composition of hepatic triglycerides in Reye's syndrome: implications for hepatic desaturase abnormalities. Clin.Chim.Acta **204:** 167-177.

Kopteva, L. A. and Dmitrieva, N. M. (1966). Concentration and nucleotide composition of nucleic acids in different parts of the dog heart during exclusion of extracardial parasympathetic influences. Biull Eksp Biol Med **61:** 47-51.

Mackenzie, B., Ahmed, A., and Rennie, M. J. (1993). Muscle Amino Acid Metabolism. *In* "Mammalian Amino Acid Transport. Mechanism and Control" (S. Michael, Ed.), pp. 195-226, Kilberg & Dieter Haussinger.

Pouw, E. M., Schols, A. M., Deutz, N. E., and Wouters, E. F. (1998). Plasma and muscle amino acid levels in relation to resting energy expenditure and inflammation in stable chronic obstructive pulmonary disease

31. Am.J Respir.Crit Care Med. **158:** 797-801.

Rabinowitz, J. L., Baker, D. G., Villanueva, T. G., Asanza, A. P., and Capuzzi, D. M. (1992). Liver lipid profiles of adults taking therapeutic doses of aspirin. Lipids **27:** 311-314.

Raclot, T., Langin, D., Lafontan, M., and Groscolas, R. (6-15-1997). Selective release of human adipocyte fatty acids according to molecular structure. Biochem.J **324 ( Pt 3):** 911-915.

Sheikh, K., Forster, J., and Nielsen, L. K. (2005). Modeling hybridoma cell metabolism using a generic genome-scale metabolic model of Mus musculus. Biotechnol Prog. **21:** 112-121.

Teufel, A., Weinmann, A., Krupp, M., Budinger, M., and Galle, P. R. (3-15-2007). Genome-wide analysis of factors regulating gene expression in liver. Gene **389:** 114-121.

THOMAS, L. W. (1962). The chemical composition of adipose tissue of man and mice. Q.J Exp.Physiol Cogn Med Sci **47:** 179-188.

Triguero, A., Barber, T., Garcia, C., Puertes, I. R., Sastre, J., and Vina, J. R. (1997). Liver intracellular L-cysteine concentration is maintained after inhibition of the trans-sulfuration pathway by propargylglycine in rats. Br.J Nutr. **78:** 823-831.
