# Supplementary material for: A multi-tissue type genome-scale metabolic network for analysis of whole-body systems physiology
Source: BMC Syst Biol. 2011 Oct 31;5:180. doi: 10.1186/1752-0509-5-180 (PMC3219569; doi:10.1186/1752-0509-5-180)
Supplement: Additional file 4 — QC/QA Tests and Futile Cycle Tests. Quality control tests to validate universal and tissue specific functionality and model consistency [file 1752-0509-5-180-S4.DOC]

# Universal Functional and Model Consistency Testing

Model consistency testing was the first step in the process of transforming the reconstructed metabolic networks into functional models. The process involved the identification of futile cycles and ensuring that the production of principle metabolites common to all three cellular models was possible. The production of principle metabolites is discussed as part of the production of precursor metabolites, amino acids, lipids, nucleotides, glycogen and cholesterol. These tests represented the most essential metabolic functions that are common to the three cell types. Biomass equations, although different for each cell type, were included in the precursor metabolite analyses because the biomass equations represented a common metabolic function that is essential for each cell type.

## Futile Cycle Tests

A futile cycle is a metabolic cycle for which the net balance of contributing reactions consists solely of the dissipation of energy [Portais et al., FEMS Microbiol Rev (2002)]. In nature futile cycles result in an energy loss from the system in the form of heat. However, in a stoichiometric network such cycles have the capacity to freely produce energy containing compounds if the reversibility of the participating reactions is not constrained. Futile cycles that could generate free energy were tested for by closing all input and output exchanges in a single cell type networks, adding demand reactions for each high energy metabolite, and either running a flux variability analysis or single optimization for each case. In addition to the production of high energy metabolites, the production of proton gradients between different sub-cellular compartments was also tested. A table indicating the reactions used and the metabolites tested is shown in Table 1.

**Table 1.** Futile Cycle Tests and Simulations

| **Optimized Reaction** | **Reaction Equation** | **Tested Metabolite** |
| --- | --- | --- |
| **ATPM** | [c] : atp + h2o --> adp + h + pi | ATP, cytosol |
| **DM_atp(g)** | [g] : atp + h2o --> adp + h + pi | ATP, golgi |
| **DM_atp(l)** | [l] : atp + h2o --> adp + h + pi | ATP, lysosome |
| **DM_atp(m)** | [m] : atp + h2o --> adp + h + pi | ATP, mitochondria |
| **DM_atp(r)** | [r] : atp + h2o --> adp + h + pi | ATP, endoplasmic reticulum |
| **DM_atp(x)** | [x] : atp + h2o --> adp + h + pi | ATP, peroxisome |
| **DM_fadh2(c)** | [c] : fadh2 --> fad + (2) h | FADH2, cytosol |
| **DM_fadh2(m)** | [m] : fadh2 --> fad + (2) h | FADH2, mitochondria |
| **DM_fadh2(x)** | [x] : fadh2 --> fad + (2) h | FADH2, peroxisome |
| **DM_h(~)** | [bl] : h --> | H+, blood |
| **DM_h(g)** | [g] : h --> | H+, golgi |
| **DM_h(l)** | [l] : h --> | H+, lysosome |
| **DM_h(m)** | [m] : h --> | H+, mitochondria |
| **DM_h(r)** | [r] : h --> | H+, endoplasmic reticulum |
| **DM_hc** | [c] : h --> | H+, cytosol |
| **DM_hx** | [x] : h --> | H+, peroxisome |
| **DM_nadh(c)** | [c] : nadh --> h + nad | NADH, cytosol |
| **DM_nadh(m)** | [m] : nadh --> h + nad | NADH, mitochondria |
| **DM_nadh(r)** | [r] : nadh --> h + nad | NADH, endoplasmic reticulum |
| **DM_nadph(c)** | [c] : nadph --> h + nadp | NADPH, cytosol |
| **DM_nadph(g)** | [g] : nadph --> h + nadp | NADPH, golgi |
| **DM_nadph(l)** | [l] : nadph --> h + nadp | NADPH, lysosome |
| **DM_nadph(m)** | [m] : nadph --> h + nadp | NADPH, mitochondria |
| **DM_nadph(r)** | [r] : nadph --> h + nadp | NADPH, endoplasmic reticulum |
| **DM_nadph(x)** | [x] : nadph --> h + nadp | NADPH, peroxisome |

Some changes were made to the adipocyte, hepatocyte and myocyte networks during this phase of testing. The following list includes reactions that were either replaced or deleted to avoid the presence of futile cycles in the myocyte network that were based on simulation results and supported by experimental evidence. The mitochondrial oxaloacetate transporter OAtrm was replaced with the mitochondrial malate/oxaloacetate shuttle MALOAtm [Palmieri, Pflugers Arch (2004)]. The mitochondrial isocitrate dehydrogenase reaction ICDHyrm was changed to be irreversible ICDHym, since this represents its activity in physiological growth conditions [Sazanov & Jackson, FEBS Letters (1994)]. The reversible alcoholdehydroenase reactions ALCD2x and ALCD2y were changed to their respective irreversible forms ALCD2if and ALCD2yf, since ethanol cannot be produced in human body. The cytosolic 3-hydroxybutyrate dehydrogenase reaction HBUTD was removed from the myocyte model, since no cytosolic form of this enzyme was found to be present neither in heart nor in skeletal muscle tissue [Watson and Lindsay, Biochem J (1972)]. The reversible aspartate/glutamate mitochondrial shuttle reaction ASPGLUm was changed to be irreversible with GACm, based on the transporter study under physiologicval conditions [Palmieri et al., EMBO J (2001)]. The mitochondrial NAD(P) transhydrogenase reversible reaction THD1m was changed to be irreversible using the reaction THD1im as well based on experimental evidences under physiological conditions [Arkblad et al., Comp Biochem Physiol B Biochem Mol Biol (2002); Hatefi and Yamaguchi, FASB J (1996)]. The mitochondrial alanine aminotransferase reaction and the mitochondrial alanine transporter ALAtm were both removed, based on the evidence that the mitochondrial alanine transaminase is present only on gluconeogenic tissues [DeRosa and Swick, J Biol Chem (1975)] and that there were no other alanine reactions in mitochondria in myocyte network. The reversible mitochondrial cysteinesulfinate/aspartate mitochondrial shuttle reaction 3SALAASPtm was replaced with the corresponding irreversible reaction 3SALAASPtim to avoid the presence of futile cycles.

## Precursor Metabolite Tests

Precursor metabolites are used in the central metabolic pathways such as glycolysis and the TCA cycle. Production of these compounds from glucose is required to validate that these pathways are functioning properly within the network. In total, the production of 12 precursor metabolites was tested in each network. The networks were allowed to consume a finite amount of glucose and all other organic compounds were constrained to be efflux only. Transport of inorganic compounds such as oxygen, carbon dioxide, phosphate, and other ions was unconstrained. A list of the reactions used and the metabolites tested for is included in Table 2.

**Table 2.** Precursor Metabolite Tests and Simulations

| **Optimized Reaction** | **Reaction Equation** | **Tested Metabolite** |
| --- | --- | --- |
| **DM_3pg(c)** | [c] : 3pg --> | 3-phospho-D-glycerate, cytosol |
| **DM_accoa1(m)** | [m] : accoa --> coa | acetyl-CoA, mitochondria |
| **DM_akg(m)** | [m] : akg --> | 2-oxoglutarate, mitochondria |
| **DM_e4p(c)** | [c] : e4p --> | 4-phospho-D-erythrose, cytosol |
| **DM_f6p(c)** | [c] : f6p --> | D-fructose 6-phosphate, cytosol |
| **DM_g3p(c)** | [c] : g3p --> | D-glyceraldehyde 3-phosphate, cytosol |
| **DM_g6p(c)** | [c] : g6p --> | D-glucose 6-phosphate, cytosol |
| **DM_oaa(m)** | [m] : oaa --> | oxaloacetate, mitochondria |
| **DM_pep(c)** | [c] : pep --> | phosphoenolpyruvate, cytosol |
| **DM_pyr(c)** | [c] : pyr --> | pyruvate, cytosol |
| **DM_r5p(c)** | [c] : r5p --> | D-ribose 5-phosphate, cytosol |
| **DM_succoa(m)** | [m] : succoa --> coa | succinyl-CoA, mitochondrial |

## Amino Acid Production Tests

Amino acid production was an important process for each network as these compounds were used as part of the cell specific biomass equations as well as alternative sources of energy in some of the networks. Of the 20 amino acids, there are 9 essential amino acids that cannot be made from glucose (histidine, isoleucine, leucine, lysine, methionine, phenylalanine, threonine, tryptophan, valine), and 2 that are conditionally essential in humans. The conditionally essential amino acids are tyrosine (produced from phenylalanine) and cysteine (produced from methionine). The production of non-essential amino acids were tested for in each model by similar methods used in the precursor metabolite tests. Production of the essential amino acids was first tested for from a finite amount of glucose. After this value was found to be zero, a finite amount of the essential amino acid was provided as an influx to the blood in addition to the glucose so as to demonstrate that the network was capable of taking up the amino acid. The same methodology was applied to the conditionally essential amino acids, except that a finite amount of the precursor amino acid was given to the blood instead of the conditionally essential amino acid. In all cases demand for the amino acid was derived from the cytosol. The reactions and metabolites tested are included in Table 3.

**Table 3.** Amino Acid Production Tests and Simulations

| **Optimized Reaction** | **Reaction Equation** | **Amino Acid** |
| --- | --- | --- |
| **DM_ala-L(c)** | [c] : ala-L --> | Alanine |
| **DM_arg-L(c)** | [c] : arg-L --> | Arginine |
| **DM_asn-L(c)** | [c] : asn-L --> | Asparagine |
| **DM_asp-L(c)** | [c] : asp-L --> | Aspartate |
| **DM_cys-L(c)** | [c] : cys-L --> | Cysteine† |
| **DM_gln-L(c)** | [c] : gln-L --> | Glutamine |
| **DM_glu-L(c)** | [c] : glu-L --> | Glutamate |
| **DM_gly(c)** | [c] : gly --> | Glycine |
| **DM_his-L(c)** | [c] : his-L --> | Histidine* |
| **DM_ile-L(c)** | [c] : ile-L --> | Isoleucine* |
| **DM_leu-L(c)** | [c] : leu-L --> | Leucine* |
| **DM_lys-L(c)** | [c] : lys-L --> | Lysine* |
| **DM_met-L(c)** | [c] : met-L --> | Methionine* |
| **DM_phe-L(c)** | [c] : phe-L --> | Phenylalanine* |
| **DM_pro-L(c)** | [c] : pro-L --> | Proline |
| **DM_ser-L(c)** | [c] : ser-L --> | Serine |
| **DM_thr-L(c)** | [c] : thr-L --> | Threonine* |
| **DM_trp-L(c)** | [c] : trp-L --> | Tryptophan* |
| **DM_tyr-L(c)** | [c] : tyr-L --> | Tyrosine† |
| **DM_val-L(c)** | [c] : val-L --> | Valine* |

* indicates an essential amino acid

† Indicates a conditionally essential amino acid

## Lipid Production Tests

Lipids are important components in the membranes of all sub-cellular organelles as well as the plasma membrane and other vesicles. The metabolic pathways for lipid synthesis were first tested by simulating the production of each fatty acid found in the specific cell types. The production of specific lipids was tested later since lipids are primarily comprised of fatty acids. Non-essential fatty acids can be made from glucose and their production was validated in each cell type in this way. There are two classes of essential and conditionally essential fatty acids. One class is n-3 and the other is n-6 fatty acids. All of the n-3 fatty acids can be produced from linolenic acid and all of the n-6 fatty acids can be produced from linoleic acid. Thus, linolenic acid and linoleic acid are essential fatty acids while the remaining n-6 and n-3 fatty acids are conditionally essential. The tests for the production of the fatty acids were preformed similarly to the amino acid production tests. The reactions and metabolites tested are included in Table 4.

The adipocyte did not initially pass all of the fatty acid production tests. The network was incapable of producing pentadecanoate (C15:0) and heptadecenoate (C17:1, n-8) from glucose. The problem was traced to a reversibility issue with the mitochondrial propionyl-CoA carboxylase reaction PPCOACm. Some evidence was found to support that the reaction is reversible *in vivo* [Reszko et al., J Biol Chem (2003); Kishimoto et al., J Lipid Res (1973)]. Replacing the irreversible reaction PPCOACm with the reversible form, PPCOACrm, resolved both of these problems.

**Table 4.** Fatty Acid Production Tests and Simulations

| **Optimized Reaction** | **Reaction Equation** | **Fatty Acid** |
| --- | --- | --- |
| **DM_dcshea3(c)** | [c] : dcshea3 --> | Docosahexaenoic acid (C22:6, n-3) |
| **DM_dcspea_3(c)** | [c] : dcspea3 --> | Clupanodonic acid (C22:5, n-3) |
| **DM_dcspea_6(c)** | [c] : dcspea6 --> | Docosapentaenoic acid (C22:5, n-6) |
| **DM_ddca(c)** | [c] : ddca --> | Lauric acid (C12:0) |
| **DM_ecsa(c)** | [c] : ecsa --> | Eicosanoic acid (C20:0) |
| **DM_ecsdea9(c)** | [c] : ecsdea9 --> | Eicosadienoic acid (C20:2, n-9) |
| **DM_ecsea9(c)** | [c] : ecsea9 --> | Eicosenoic acid (C20:1, n-9) |
| **DM_ecspea3(c)** | [c] : ecspea3 --> | Timnodonic acid (C20:5, n-3) |
| **DM_ecstea6(c)** | [c] : ecstea6 --> | Eicosatrienoic acid (C20:3, n-6) |
| **DM_ecstea9(c)** | [c] : ecstea9 --> | Mead acid (C20:3, n-9) |
| **DM_ecsttea6(c)** | [c] : ecsttea6 --> | Arachidonic acid (C20:4, n-6) |
| **DM_fa13(c)** | [c] : ocdca --> | Stearic acid (C18:0) |
| **DM_fa8(c)** | [c] : hdca --> | Palmitic acid (C16:0) |
| **DM_hdcea7(c)** | [c] : hdcea7 --> | Hexadecenoic acid (C16:1, n-7) |
| **DM_hpdca(c)** | [c] : hpdca --> | Margaric acid (C17:0) |
| **DM_ilnlc(c)** | [c] : ilnlc --> | Isolinoleic acid (C18:2, n-9) |
| **DM_ocdcea9(c)** | [c] : ocdcea9 --> | Oleic acid (C18:1, n-9) |
| **DM_ocdctra3(c)** | [c] : ocdctra3 --> | Linolenic acid (C18:3, n-3)* |
| **DM_ocdctra6(c)** | [c] : ocdctra6 --> | Gamolenic acid (C18:3, n-6) |
| **DM_ocddea6(c)_hep** | [c] : ocddea6 --> | Linoleic acid (C18:2, n-6)* |
| **DM_ocsttea6(c)** | [c] : ocsttea6 --> | Adrenic acid (C22:4, n-6) |
| **DM_ptdca(c)** | [c] : ptdca --> | Pentadecanoic acid (C15:0) |
| **DM_ttdca(c)** | [c] : ttdca --> | Myristic acid (C14:0) |
| **DM_ttdcea7(c)** | [c] : ttdcea7 --> | Tetradecenoic acid (C14:1, n-7) |
| **DM_dcsea9(c)** | [c] : dcsea9 --> | Docosenoic acid (C22:1, n-9) |
| **DM_ecsea11(c)** | [c] : ecsea11 --> | Eicosenoic acid (C20:1, n-11) |
| **DM_hdcea9(c)** | [c] : hdcea9 --> | Hexadecenoic acid (C16:1, n-9) |
| **DM_hpdcea8(c)** | [c] : hpdcea8 --> | Heptadecenoic acid (C17:1, n-8) |
| **DM_ocdcea5(c)** | [c] : ocdcea5 --> | Octadecenoic acid (C18:1, n-5) |
| **DM_ocdcea7(c)** | [c] : ocdcea7 --> | Octadecenoic acid (C18:1, n-7) |
| **DM_ttdcea5(c)** | [c] : ttdcea5 --> | Myristoleic acid (C14:1, n-5) |
| **DM_dcsa(c)** | [c] : dcsa --> | Behenic acid (n-C22:0) |
| **DM_ecsdea6(c)** | [c] : ecsdea6 --> | Eicosadienoic acid (C20:2, n-6) |
| **DM_hpdca(c)** | [c] : hpdca --> | Margaric acid (C17:0) |

* indicates an essential fatty acid

Validation of the myocyte network included simulating the ATP production from one mole of octadecenoate (C18:1), palmitate (C16:0) and pentadecanoate (C15:0) (Table 2). To demonstrate how each of these fatty acids could be catabolized to produce energy, the influx of all other carbon sources including glucose was constrained to zero and internal demand for cytosolic ATP was maximized. The myocyte simulations demonstrated that a unit of proton per fatty acid was required to balance fatty acyl-CoA formation in the network. The proton demand was also identified and supplied to the myocyte metabolic network. The liable explanation for proton demand is the role of the proton electrochemical gradient across the inner membrane to energize the long-chain fatty acid transport apparatus. This has been observed in E.coli and has been shown to be required for optimal fatty acid transport.

The energy (ATP) production was calculated to be 120.75 mol ATP/ mol of octadecenoate (C18:1), 108 mol ATP/ mol of palmitate (C16:0) and 100.25 mol ATP/ mol of pentadecanoate (C15:0). The calculated ATP values are slightly different between two models. Published experimental data and previous reconstructions of mitochondrial metabolism match results calculated in Phase I myocyte model and report that 106 mol of ATP is produced from one mole of palmitate, when the P/O ratio is 2.5.

**Table 5.** Simulated energy production from single fatty acids in myocyte model.

| Fatty Acid | Abbreviation | Myocyte  Model |
| --- | --- | --- |
| Octadecenoate | C18:1 | 120.75 |
| Palmitate | C16:0 | 108 |
| Pentadecanoate | C15:0 | 100.25 |

Further evaluation of the myocyte metabolic network allowed for identification of the metabolic difference, which caused a variation of 2 ATP mols. In the reconstructed myocyte metabolic model, reactions that are catalyzed by the NADP dependent malic enzyme are included to be reversible, based on the previous experimental evidence generated using various types of mammalian cell types and tissues. In this case, cytosolic NADP-dependent malic enzyme performs in the reverse direction allowing for transfer of reducing equivalents from the cytosol into mitochondria via the shuttle mechanism, which consequently contributes to additional production of ATP.

After fatty acid production had been verified for each cell type, the production of cell specific lipids was tested. Cell specific lipids include macromolecules such as triacylglycerol, phosphatidylserine, ceramide and phosphatidate. Each of these compounds has a slightly different lipid composition. The reactions and metabolites that were examined are included in Table 6.

The adipocyte did not initially pass all of the lipid production tests. The reaction FAVGPA_Hsa_Adp had to be added to the model in order to allow the production of the adipocyte specific monoacylglycerol species. The reaction allows for an averaged fatty acid to be decomposed into its specific fatty acid components and is thus required for modeling purposes only.

**Table 6.** Lipid Production Tests and Simulations

| **Optimized Reaction** | **Reaction Equation** | **Network** | **Lipid Type** |
| --- | --- | --- | --- |
| **DM_12dgr_Hsa_Hep** | [c] : 12dgr_Hsa_Hep --> | Hepatocyte | 1,2-Diacylglycerol |
| **DM_1ag3p_Hsa_Hep(c)** | [c] : 1ag3p_Hsa_Hep --> | Hepatocyte | 1-Acyl-sn-glycerol 3-phosphate |
| **DM_1aglycpc_Hsa_Hep(c)** | [c] : 1aglycpc_Hsa_Hep --> | Hepatocyte | 1-Acyl-sn-glycero-3-phosphocholine |
| **DM_cdpdag_Hsa_Hep(c)** | [c] : cdpdag_Hsa_Hep --> | Hepatocyte | CDPdiacylglycerol |
| **DM_cer_Hsa_Hep(c)** | [c] : cer_Hsa_Hep --> | Hepatocyte | Ceramide |
| **DM_cholse_Hsa_Hep(c)** | [c] : cholse_Hsa_Hep --> | Hepatocyte | Cholesterol ester |
| **DM_clpn_Hsa_Hep(c)** | [c] : clpn_Hsa_Hep --> | Hepatocyte | Cardiolipin |
| **DM_dcer_Hsa_Hep(c)** | [c] : dcer_Hsa_Hep --> | Hepatocyte | Dihydroceramide |
| **DM_fa_avg_Hsa_Hep(c)** | [c] : fa_avg_Hsa_Hep --> | Hepatocyte | Averaged fatty acid |
| **DM_facoa_avg_Hsa_Hep(c)** | [c] : (0.001) facoa_avg_Hsa_Hep --> coa | Hepatocyte | Averaged fatty-acyl CoA |
| **DM_pa_Hsa_Hep(c)** | [c] : pa_Hsa_Hep --> | Hepatocyte | Phosphatidate |
| **DM_pc_Hsa_Hep(c)** | [c] : pc_Hsa_Hep --> | Hepatocyte | Phosphatidylcholine |
| **DM_pe_Hsa_Hep(c)** | [c] : pe_Hsa_Hep --> | Hepatocyte | Phosphatidylethanolamine |
| **DM_pg_Hsa_Hep(m)** | [m] : pg_Hsa_Hep --> | Hepatocyte | Phosphatidylglycerol |
| **DM_pgp_Hsa_Hep(m)** | [m] : pgp_Hsa_Hep --> | Hepatocyte | Phosphatidylglycerophosphate |
| **DM_pino_Hsa_Hep(c)** | [c] : pino_Hsa_Hep --> | Hepatocyte | Phosphatidyl-1D-myo-inositol |
| **DM_ps_Hsa_Hep(c)** | [c] : ps_Hsa_Hep --> | Hepatocyte | Phosphatidylserine |
| **DM_sphgmy_Hsa_Hep(c)** | [c] : sphgmy_Hsa_Hep --> | Hepatocyte | Sphingomyelin |
| **DM_triglyc_Hsa_Hep** | [c] : triglyc_Hsa_Hep --> | Hepatocyte | Triglyceride |
| **DM_cer_Adp(c)** | [c] : cer_Hsa_Adp --> | Adipocyte | Ceramide |
| **DM_clpn_Hsa_Adp(c)** | [c] : clpn_Hsa_Adp --> | Adipocyte | Cardiolipin |
| **DM_dcer_Hsa_Adp(c)** | [c] : dcer_Hsa_Adp --> | Adipocyte | Dihydroceramide |
| **DM_mglyc_Hsa_Adp(c)** | [c] : mglyc_Hsa_Adp --> | Adipocyte | Monoacylglycerol |
| **DM_sphgmy_Hsa_Adp(c)** | [c] : sphgmy_Hsa_Adp --> | Adipocyte | Sphingomyelin |
| **DM_12dgr_Hsa_Adp(c)** | [c] : 12dgr_Hsa_Adp --> | Adipocyte | 1,2-Diacylglycerol |
| **DM_1ag3p_HS_Adp(c)** | [c] : 1ag3p_Hsa_Adp --> | Adipocyte | 1-Acyl-sn-glycerol 3-phosphate |
| **DM_cdpdag_Hsa_Adp(c)** | [c] : cdpdag_Hsa_Adp --> | Adipocyte | CDPdiacylglycerol |
| **DM_fa_avg_Hsa_Adp(c)** | [c] : fa_avg_Hsa_Adp --> | Adipocyte | Averaged fatty acid |
| **DM_facoa_avg_Hsa_Adp(c)** | [c] : (0.001) facoa_avg_Hsa_Adp --> coa | Adipocyte | Averaged fatty-acyl CoA |
| **DM_pa_Hsa_Adp(c)** | [c] : pa_Hsa_Adp --> | Adipocyte | Phosphatidate |
| **DM_pc_Hsa_Adp(c)** | [c] : pc_Hsa_Adp --> | Adipocyte | Phosphatidylcholine |
| **DM_pe_HS_Adp(c)** | [c] : pe_Hsa_Adp --> | Adipocyte | Phosphatidylethanolamine |
| **DM_pg_Hsa_Adp(m)** | [m] : pg_Hsa_Adp --> | Adipocyte | Phosphatidylglycerol |
| **DM_pgp_Hsa_Adp(m)** | [m] : pgp_Hsa_Adp --> | Adipocyte | Phosphatidylglycerophosphate |
| **DM_pino_Hsa_Adp(c)** | [c] : pino_Hsa_Adp --> | Adipocyte | Phosphatidyl-1D-myo-inositol |
| **DM_ps_Hsa_Adp(c)** | [c] : ps_Hsa_Adp --> | Adipocyte | Phosphatidylserine |
| **DM_triglyc_Hsa_Adp(c)** | [c] : triglyc_Hsa_Adp --> | Adipocyte | Triglyceride |
| **DM_cholse_Hsa_Myc(c)** | [c] : cholse_Hsa_Myc --> | Myocyte | Cholesterol ester |
| **DM_clpn_Hsa_Myc** | [c] : clpn_Hsa_Myc --> | Myocyte | Cardiolipin |
| **DM_triglyc_Hsa_Myc** | [c] : triglyc_Hsa_Myc --> | Myocyte | Triglyceride |
| **DM_12dgr_Hsa_Myc** | [c] : 12dgr_Hsa_Myc --> | Myocyte | 1,2-Diacylglycerol |
| **DM_mglyc_Hsa_Myc** | [c] : mglyc_Hsa_Myc --> | Myocyte | Monoacylglycerol |
| **DM_pe_Hsa_Myc(c)** | [c] : pe_Hsa_Myc --> | Myocyte | Phosphatidylethanolamine |
| **DM_pino_Hsa_Myc(c)** | [c] : pino_Hsa_Myc --> | Myocyte | Phosphatidyl-1D-myo-inositol |
| **DM_pc_Hsa_Myc(c)** | [c] : pc_Hsa_Myc --> | Myocyte | Phosphatidylcholine |
| **DM_ps_Hsa_Myc(c)** | [c] : ps_Hsa_Myc --> | Myocyte | Phosphatidylserine |
| **DM_sphgmy_Hsa_Myc** | [c] : sphgmy_Hsa_Myc --> | Myocyte | Sphingomyeline |
| **DM_clpn_Hsa_Myc(m)** | [m] : clpn_Hsa_Myc --> | Myocyte | Cardiolipin |

## Nucleotide Production Tests

The three reconstructed cell type networks are capable of producing all the nucleotide bases used in RNA and DNA from glucose. They are also capable of producing the mono- and tri-phosphorylated forms of these bases. Nucleotide production was tested using a finite amount of glucose under conditions similar to that of the precursor metabolite tests. A table indicating the reactions used and the metabolites tested is shown in Table 7.

**Table 7.** Nucleotide Production Tests and Simulations

| **Optimized Reaction** | **Reaction Equation** | **Nucleotide** |
| --- | --- | --- |
| **DM_atp** | [c] : atp --> | ATP |
| **DM_ctp(c)** | [c] : ctp --> | CTP |
| **DM_datp(c)** | [c] : datp --> | dATP |
| **DM_dctp(c)** | [c] : dctp --> | dCTP |
| **DM_dgtp(c)** | [c] : dgtp --> | dGTP |
| **DM_dttp(c)** | [c] : dttp --> | dTTP |
| **DM_gtp(c)** | [c] : gtp --> | GTP |
| **DM_utp(c)** | [c] : utp --> | UTP |

## Glycogen and Cholesterol Production Tests

Glycogen is an important energy storage molecule present in all 3 cell types. Glycogen can be rapidly broken down into glucose-6-P and enter glycolysis, providing the cell with an immediate source of energy when blood glucose is scarce. Cholesterol is an essential component of lipid membranes and it serves to regulate the permeability and fluidity of such membranes as well as being the precursor for many steroid hormones. Each of the three reconstructed networks is capable of producing cholesterol from glucose. Glycogen and cholesterol production were tested in each cell type in a manner similar to the precursor metabolite tests. Table 8 provides a brief overview of the reactions used to test the networks.

**Table 8.** Glycogen and Cholesterol Production Tests and Simulations

| **Optimized Reaction** | **Reaction Equation** | **Network** | **Tested Metabolite** | **Mol metabolite / mol Glucose** |
| --- | --- | --- | --- | --- |
| **DM_glycogen(c)** | [c] : glycogen --> | Hepatocyte | Glycogen, cytosol | 0.942 |
| **DM_chsterol(c)** | [c] : chsterol --> | Hepatocyte | Cholesterol, cytosol | 0.092 |
| **DM_glycogen(c)** | [c] : glycogen --> | Myocyte | Glycogen, cytosol | 0.942 |
| **DM_chsterol(c)** | [c] : chsterol --> | Myocyte | Cholesterol, cytosol | 0.092 |
| **DM_glycogen(c)** | [c] : glycogen --> | Adipocyte | Glycogen, cytosol | 0.942 |
| **DM_chsterol(c)** | [c] : chsterol --> | Adipocyte | Cholesterol, cytosol | 0.092 |

When first tested, the adipocyte network could not produce cholesterol. The problem was due to the need to dissipate protons that accumulate in the endoplasmic reticulum (ER) from the cholesterol biosynthetic pathway. The hepatocyte and myocyte networks circumvented this problem through the use of glucose-6-posphatase (G6PASEer) in the ER and the ER pyruvate transporter. Since the pyruvate transporter was proton coupled and the G6PASEer reaction was independent of the cholesterol production pathway, the correct number of protons could escape the ER based on flux through these reactions. There was some evidence for the phosphatase activity in adipocytes in the ER [Watanabe et al., Anat Rec 1987]. However, it is unlikely that flux through this pathway is representative of cellular physiology when the cells are producing cholesterol since there are many ER transporters that have not been included in the latest network reconstructions. Since there is currently no other way to facilitate a proton efflux from the ER, the inclusion of the G6PASEer reaction remains the best solution for now.

## Biomass Functional Tests

The biomass reaction for each cell type was based on the published molecular content of each representative cell type. In each case, the cell was given a finite amount of glucose, essential amino acids, and fatty acids. Choline was not constrained as it is an essential vitamin and cannot be metabolized by any of the networks while all other organic compounds were constrained to be a unidirectional efflux from the blood. Inorganic compounds and ions were not constrained. The biomass equation was added to the model and maximized as part of a single optimization simulation. Table 9 provides a brief overview of the reactions used in each case.

**Table 9.** Biomass Functional Tests and Simulations

| **Reaction Abbreviation** | **Network** | **Maximal Biomass Flux Value** |
| --- | --- | --- |
| **Biomass_Hsa_Hep_26GAM** | Hepatocyte | 1.164 |
| **Biomass_Hsa_Adp_1GAM** | Adipocyte | 0.712 |
| **Biomass_Hsa_Myc_35GAM** | Myocyte | 1.225 |

# Cell Specific Functional Testing

Each reconstructed cellular network contains metabolic functions that are specific to the physiology of the distinct cell type. For instance bile salts are only synthesized in the hepatocyte, and only myocytes can convert chemical energy into the mechanical energy of a muscle contraction. Prior to the multi-cell simulation the divergent cell specific metabolic functions of each individual network were examined separately to characterize their behavior. This was an essential prerequisite to understanding the integrated functional characteristics of the different cell types.

## Hepatocyte Functional Tests

The hepatocyte has a wide range of biochemical functions in the human body. Hepatocytes are responsible for the synthesis of glucose to maintain the blood glucose level. They also synthesize bile salts for the digestion of comestibles, produce ketone bodies that can be used as fuel for nervous tissue, and they can metabolize alternative sugars such as galactose and mannose. The following section describes all the functional tests that were preformed for the hepatocyte.

### Gluconeogenesis

The hepatocyte network was first tested for its ability to produce glucose from a variety of gluconeogenic substrates. Gluconeogenic substrates include all of the common amino acids, lactate, glycerol, and pyruvate. Simulations were devised for testing several different amino acids that were important for multi-cellular cycles, lactate, glycerol, and pyruvate. Metabolic simulation initially showed that the network was incapable of producing glucose from any substrate on its own. The problem was traced to the need to export inorganic phosphate from the endoplasmic reticulum (ER) lumen. In the hepatocyte the irreversible phosphorylation of glucose is circumvented in the ER lumen by decoupling the step with ATP hydrolysis. The resulting inorganic phosphate is then transported out of the ER by a proton coupled symport transport protein. Since the network had no way of producing hydrogen in the ER lumen, the ATP coupled proton transporter vacuolar ATPase was used to enable proton transport into the ER lumen. Although evidence was found for the utilization of ATP in the active regulation of pH in hepatocyte organelles [Jefferies et al., Arch Biochem Biophys (2008); Wadsworth and van Rossum, J Membr Biol (1994)], the specific gene was not assigned because no evidence was found describing the particular gene. Additional tests were performed using several different fatty acids, ethanol, and acetate. These metabolites are not gluconeogenic substrates and the network confirmed that glucose could not be produced from them. These simulations have been omitted from this report.

**Table 10.** Gluconeogenic Simulations

| **Optimized Reaction** | **Reaction Equation** | **Gluconegenic Substrate** | **Max glucose / substrate flux** |
| --- | --- | --- | --- |
| **EX_glc-D(bl)** | [bl] : glc-D <==> | Glycerol | 0.5 |
| **EX_glc-D(bl)** | [bl] : glc-D <==> | L-lactate | 0.424 |
| **EX_glc-D(bl)** | [bl] : glc-D <==> | Alanine | 0.424 |
| **EX_glc-D(bl)** | [bl] : glc-D <==> | Glutamine | 0.5 |
| **EX_glc-D(bl)** | [bl] : glc-D <==> | Pyruvate | 0.354 |
| **EX_glc-D(bl)** | [bl] : glc-D <==> | Threonine | 0.5 |

### Ketogenesis

Hepatocytes are capable of producing the ketone bodies (KB), acetoacetate and β-hydroxybutyrate when there are low concentrations of carbohydrates in the blood. In this state, ketone bodies are produced from the β-oxidation of fatty acids. The ketogenesis pathways were not included in Recon 1 and were reviewed and added later as a part of later research. After the pathways were added, the production of acetoacetate and β-hydroxybutyrate were tested using eicosenoate (C20:1, n-9) as a model fatty acid. The simulations can be found in Table 11. In addition to the physiologically correct pathway of ketogenesis, the hepatocyte network was also found to be capable of producing ketone bodies from glucose. This is not observed physiologically due to the down regulation of ketogenesis when large quantities of carbohydrates are present [Stipanuk, 2000]. This simulation was intentionally omitted from this report.

**Table 11.** Ketogenic Simulations

| **Optimized Reaction** | **Reaction Equation** | **Metabolite** | **Max KB production / eicosenoate (C20:1, n-9)** |
| --- | --- | --- | --- |
| **EX_acac(bl)** | [bl] : acac <==> | Acetoacetate | 5 |
| **EX_3hbut-R(bl)** | [bl] : 3hbut-R <==> | β-hydroxybutyrate | 5 |

### Alternative Sugar Metabolism

Although glucose is the primary energy source for all the cells in the human body, hepatocytes are capable of using alternative sugars as energy sources [Stipanuk, 2000]. Alternative pathways for fructose, galactose, and mannose were reviewed and added to the hepatocyte network since these were not originally included in the reconstructed network. The tests showed that the same amount of ATP could be produced on a molar basis from glucose, galactose and mannose, and fructose (32.5 mol ATP/mol fructose). The simulations can be found in Table 12.

**Table 12.** Alternative Sugar Simulations

| **Optimized reaction** | **Reaction Equation** | **Tested Sugar** | **mol ATP/mol sugar** |
| --- | --- | --- | --- |
| **ATPM** | [c] : atp + h2o --> adp + h + pi | Fructose | 32.5 |
| **ATPM** | [c] : atp + h2o --> adp + h + pi | Galactose | 32.5 |
| **ATPM** | [c] : atp + h2o --> adp + h + pi | Mannose | 32.5 |

## Adipocyte Functional Tests

Adipocytes are specialized cells that store excess accumulated energy as triglycerides and other lipids. The functional testing of the adipocyte network involved demonstrating the production of lactate, glycerol, and fatty acids from glucose as well as from triacylglycerol. In both cases octadecenoate (C18:1, n-7) was used to test the production of fatty acid. The simulations are described in Table 15.

**Table 15.** Adipocyte Functional Simulations

| **Optimized Reaction** | **Reaction Equation** | **Tested Metabolites** | **Max mol product / mol substrate** |
| --- | --- | --- | --- |
| **EX_lac-L(bl)** | [bl] : lac-L <==> | Glucose to Lactate | 2.0 |
| **EX_glyc(bl)** | [bl] : glyc <==> | Glucose to Glycerol | 1.368 |
| **EX_ocdcea7(bl)** | [bl] : ocdcea7 <==> | Glucose to Octadecenoic acid (C18:1, n-7) | 0.207 |
| **EX_lac-L(bl)** | [bl] : lac-L <==> | Triacylglycerol to Lactate | 1,009.0 |
| **EX_glyc(bl)** | [bl] : glyc <==> | Triacylglycerol to Glycerol | 1,009.0 |
| **EX_ocdcea7(bl)** | [bl] : ocdcea7 <==> | Triacylglycerol to Octadecenoic acid (C18:1, n-7) | 2,528.1 |

The adipocyte was also used to compare its predicted fluxes from experimentally determined ones. Only one data set was found [Si et al., Am J Physiol Endocrinol Metab (2007)], however, the conditions that had the best carbon balance produced an infeasible result when used in the adipocyte network.

## Myocyte Functional Tests

Myocytes account for a significant portion of body mass in an average adult. They are capable of converting chemical energy into mechanical energy and facilitate movement of the body. The myocyte is capable of using glucose, branched chain amino acids, and ketone bodies, glycogen, and fatty acids as energy sources. In anaerobic conditions during an intense exercise such as a sprint, skeletal muscles will also produce significant quantities of lactate. During the postprandial phase (following a meal) myocytes will also produce alanine from glucose as part of the glucose-alanine cycle. Each of these functions was tested individually with the network. The simulations can be found from Table 16.

**Table 16.** Myocyte Functional Simulations

| **Optimized Reaction** | **Reaction Equation** | **Tested Metabolites** | **Max mol product / mol substrate** |
| --- | --- | --- | --- |
| **EX_lac-L(bl)** | [bl] : lac-L <==> | Glucose to Lactate | 2.0 |
| **EX_ala-L(bl)** | [bl] : ala-L <==> | Glucose to Alanine | 2.0 |
| **EX_gln-L(bl)** | [bl] : gln-L <==> | Glucose to Glutamine | 1.0 |
| **ATPM** | [c] : atp + h2o --> adp + h + pi | Isoleucine degradation | 33.625 |
| **ATPM** | [c] : atp + h2o --> adp + h + pi | Valine degradation | 27.125 |
| **ATPM** | [c] : atp + h2o --> adp + h + pi | Leucine degradation | 32.625 |
| **ATPM** | [c] : atp + h2o --> adp + h + pi | Triacylglycerol degradation | 370,593.0 |
| **ATPM** | [c] : atp + h2o --> adp + h + pi | L-lactate degradation | 15.25 |
| **ATPM** | [c] : atp + h2o --> adp + h + pi | Acetoacetate degradation | 19.0 |
| **ATPM** | [c] : atp + h2o --> adp + h + pi | β-hydroxybutyrate degradation | 21.5 |
| **EX_lac-L(bl)** | [bl] : lac-L <==> | Glycogen to Lactate | 2.0 |
| **DM_glycogen(c)** | [c] : glycogen --> | Glucose to Glycogen | 0.942 |
| **EX_ala-L(bl)** | [bl] : ala-L <==> | Isoleucine to alanine | 1.0 |
| **EX_ala-L(bl)** | [bl] : ala-L <==> | Valine to alanine | 1.0 |
| **EX_ala-L(bl)** | [bl] : ala-L <==> | Glycogen to alanine | 2.0 |
| **ATPM** | [c] : atp + h2o --> adp + h + pi | Oleic acid (C18:1, n-9) degradation | 116.375 |

To simulate muscle contraction, earlier generated burn and sham skeletal muscle tissue flux data [Banta, 2004] was used. Carbon balance and 26 specific metabolite consumption and metabolite production rates for four different datasets – (i) sham skeletal muscle, (ii) burn skeletal muscle, (iii) insulin treated sham skeletal muscle, (iv) insulin treated burn skeletal muscle – were calculated. The carbon balance closed to 104 ± 4%. Calculated specific metabolite uptake and metabolite production rates were fit to the myocyte network and four simulations representing the four different experimental conditions were generated. During the data fitting, two demand reactions for citrate and glycogen needed to be added to the model. Moreover, fluxes for two amino acids were excluded from the four simulations, based on the myocyte model features. For instance Banta’s data showed lysine production, but in the myocyte network lysine is an essential amino acid that cannot be produced. Further study is needed to understand the lysine discrepancy. Maximum contraction was calculated using fitted flux data (Table 17). When the flux data was fitted to the myocyte network, the computational analysis revealed that burn tissue, which is known to be in a hypermetabolic state, generates 9% more energy compared to sham tissue. The differences between the insulin supplemented and non-supplemented states in the control tissue was negligible with regard to maximal ATP production. In agreement to prior insulin therapy results [Banta, 2004], the simulation results showed that insulin represses burn muscle tissue wasting by 21%.

**Table 17.** Simulated energy production using Banta’s (2004) flux dataset.

| Sample | Contraction |
| --- | --- |
| Sham | 80.8 |
| Sham + Insulin | 82.0 |
| Burn | 88.1 |
| Burn + Insulin | 72.5 |

After analyzing the data from all the cell specific functional tests, the directionality of the mitochondrial pyruvate transporter was consistently found to operate as an importer of pyruvate into the mitochondria. This result is physiologically valid since the mechanism of pyruvate transport is a proton coupled symport reaction [Palmieri, Pflugers Arch (2004)] and under normal conditions there is a proton gradient that exists across the inner mitochondrial membrane. For these reasons the reversible mitochondrial pyruvate transport reaction PYRtm was changed to the irreversible form PYRtim in all the cell type networks.

**References**

1. Portais, JC and Delort, AM. Carbohydrate cycling in micro-organisms: what can (13)C-NMR tell us? *FEMS Microbiol Rev* **26**, 375-402 (2002).
2. Palmieri, F. The mitochondrial transporter family (SLC25): physiological and pathological implications. *Pflugers Arch*. **447**, 689-709 (2004).
3. Sazanov, LA and Jackson, JB. Proton-translocating transhydrogenase and NAD- and NADP- linked isocitrate dehydrogenases operate in a substrate cycle which contributes to fine regulation of the tricarboxylic acid cycle activity in mitochondria. *FEBS Lett.* **334**, 109-116 (1994).
4. Watson, HR and Lindsay, DB. 3-hydroxybutyrate dehydrogenase in tissues from normal and ketonaemic sheep. *Biochem J* **128**, 53-57 (1972).
5. Palmieri, L et al. Citrin and aralar1 are Ca(2+)-stimulated aspartate/glutamate transporters in mitochondria. *EMBO J* **20**, 5060-5069 (2001).
6. Arkblad, EL et al. Expression of proton-pumping nicotinamide nucleotide transhydrogenase in mouse, human brain and C elegans. *Comp Biochem Physiol B Biochem Mol Biol* **133**, 13-21 (2002).
7. Hatefi, Y and Yamaguchi M. Nicotinamide nucleotide transhydrogenase: a model for utilization of substrate binding energy for proton translocation. *FASEB J* **10**, 444-452 (1996).
8. DeRosa, G and Swick RW. Metabolic implications of the distribution of the alanine aminotransferase isoenzymes. *J Biol Chem*. **250**, 7961-7967 (1975).
9. Reszko, AE et al. Assessing the reversibility of the anaplerotic reactions of the propionyl-CoA pathway in heart and liver. *J Biol Chem.* **278**, 34959-34965 (2003).
10. Kishimoto, Y et al. Branched-chain and odd-numbered fatty acids and aldehydes in the nervous system of a patient with deranged vitamin B 12 metabolism. *J Lipid Res*. **14**, 69-77 (1973).
11. Watanabe, J et al. Signifiance of increase in glucose 6-phosphatase activity in brown adipose cells of cold-exposed and starved mice. *Anat Rec.* **219**, 39-44 (1987).
12. Jeffries, KC et al. Function, structure, and regulation of the vacuolar (H+)-ATPases. *Arch. Biochem. Biophys.* **476**, 33-42 (2008).
13. Wadworth, SJ and van Rossum, GD. Role of vacuolar adenosine triphosphatase in the regulation of cytosolic pH in hepatocytes. *J Membr Biol.* **142**, 21-34 (1994).
14. Stipanuk, MH. *Biochemical and physiological aspects of human nutrition*. WB Saunders Company, Philadelphia, PA (2000).
15. Si, Y et al. Flux profile and modularity analysis of time-dependent metabolic changes of de novo adipocyte formation. *Am J Physiol Endocrinol Metab* **292**, E1637-E1646 (2007).
16. Banta, S et al. Quantitative effects of thermal injury and insulin on the metabolism of the skeletal muscle using the perfused rat hindquarter preparation. *Biotechnol Bioeng* **88**, 613-629 (2004).
