# Supplementary material for: A multi-tissue type genome-scale metabolic network for analysis of whole-body systems physiology
Source: BMC Syst Biol. 2011 Oct 31;5:180. doi: 10.1186/1752-0509-5-180 (PMC3219569; doi:10.1186/1752-0509-5-180)
Supplement: Additional file 5 — Unconstrained simulations of Cori and Alanine Cycles. Additional information on simulating the metabolic interaction between the liver and muscle based on sensitivity analysis of objectives (hepatic glucose and urea production) [file 1752-0509-5-180-S5.DOC]

**Unconstrained Simulations of the Alanine and Cori Cycles**

In the main text, constraints are set on MM metabolite production (lactate and alanine) to amplify and study the efficiency and flux span of each metabolic cycle separately. Physiologically, the two cycles do not occur exclusively. To further analyze the interaction between the HM and MM, an unconstrained assessment was completed.

First, unconstrained simulations of the Cori Cycle yielded the same results as the constrained simulations. This result suggests that the most efficient method of carbon recycling is through muscle production of lactate. However, unconstrained simulations of the Alanine Cycle did not yield the same results as the complement constrained simulations. In fact, unconstrained simulations reverted to using lactate as it is more efficient to produce glucose in the liver. This is due to the fact that the objective of the simulation was glucose production, whilst the main objective of the Alanine Cycle is to remove nitrogen from the muscles. In contrast, optimizing just for urea extraction from the liver yields proper MM production and HM utilization of alanine, but no glucose is produced.

Thus, a sensitivity analysis was completed to compare the effect of glucose and urea production versus lactate and alanine usage. The objective weighting for glucose production was set to 1, while the objective weighting for urea production was varied from 0 to 3 through 300 iterations (Figure S1). At each iteration, the minimum and maximum MM production of alanine and lactate was calculated through flux variability analysis.

When glucose production is greater than urea production, lactate is the only MM produced metabolite. At a weighting of 1.66 for urea production versus a weighting of 1 for glucose production, the flux through urea and glucose production is equal. At this point, alanine utilization is possible. This makes sense as the Alanine Cycle produces urea and glucose at a 1:1 ratio. Lower urea production is not possible with alanine as the HM substrate and thus lactate is utilized to produce glucose as well as generate ATP to produce urea from other nitrogen containing amino acid sources. At a 1:1 ratio or greater, alanine usage greatly increases until at a weighting of 1.83 for urea production to a weighting of 1 for glucose, alanine is the only HM substrate. At this point glucose production is reduced to about 5% of its maximum production under lactate.

The physiological range for the Alanine cycle falls around a 1:1 ratio where both lactate and alanine are utilized. Our sensitivity analysis shows that the two cycles are not physiologically exclusive as both alanine and lactate are utilized to recycle nitrogen and carbon.


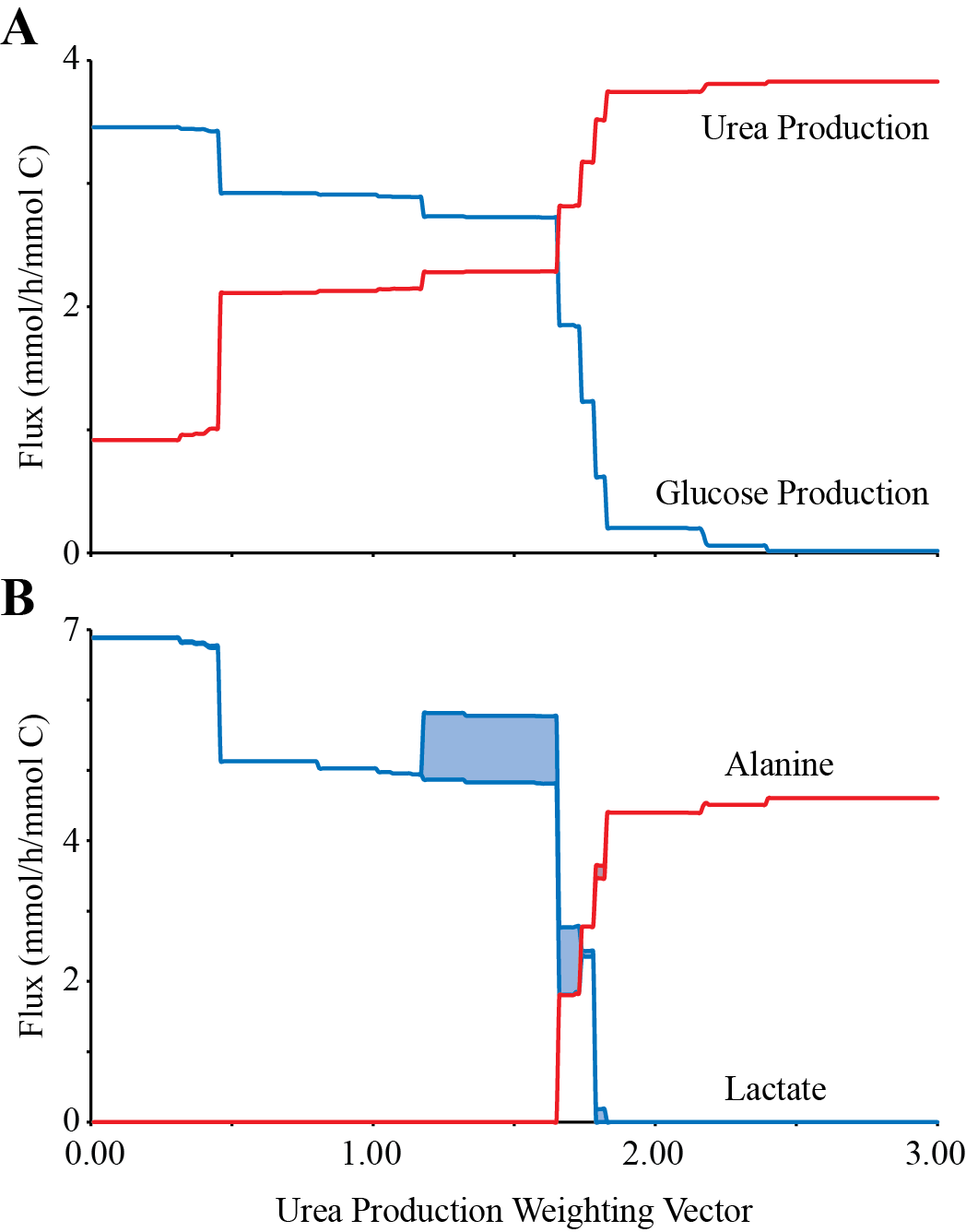


**Figure 1**: Sensitivity analysis of glucose and urea production in the HM. The urea production weighting vector was varied from 0 to 3 to vary the production of glucose and urea by the HM. **(A)** Plot of urea and glucose production as a function of the urea production weighting vector. At 1.66, the production of urea and glucose in the HM are equal. **(B)** The possible flux ranges for alanine and lactate production by the MM were also calculated by flux variability. More flexible regions occurs around a 1:1 ratio of urea to glucose HM production.
