# Supplementary material for: A multi-tissue type genome-scale metabolic network for analysis of whole-body systems physiology
Source: BMC Syst Biol. 2011 Oct 31;5:180. doi: 10.1186/1752-0509-5-180 (PMC3219569; doi:10.1186/1752-0509-5-180)
Supplement: Additional file 7 — Liver metabolic network reconstruction comparison. Comparison of HM, Recon 1, and two existing liver metabolic networks in terms of metabolites and reactions. [file 1752-0509-5-180-S7.DOC]

*Hepatic Model Comparison*

For the scope of studying multi-tissue interactions, a HM was constructed that described the central metabolism of the liver including carbon, amino acid, and fatty acid metabolism. Crucial metabolic functions of the hepatocyte were included: gluconeogenesis, ketogenesis, urea and bile production, and glycogen storage. The tissue models in this study update Recon 1 to introduce RefSeq Ids for GPR associations, rather than Entrez Gene Ids. RefSeq Ids are more specific and allow for accurate high-throughput data mapping. In addition, transporters are properly ion and charge balanced, improving upon Recon 1 transporters. Currently, two hepatic metabolic reconstructions are available [1,2]. The Jerby network is an automated reconstruction, derived from Recon 1. The Gille network was manually reconstructed including components from Recon 1 and additional hepatic metabolism.

A comparison was done between the three networks (HM, Jerby, Gille) and Recon 1, comparing unique metabolites and intracellular reactions (excluding all transporters). Comparisons for genes was not possible. This is due to the Gille network not having explicit GPR associations and the Jerby network having the same GPRs as Recon 1. Not updating the GPRs from Recon 1 is problematic when considering metabolic reactions with a GPR similar to (Gene_A OR Gene_B). If Gene_A is not present in hepatocytes but Gene_B is, a single gene deletion of Gene_B should knockout the particular metabolic reaction. However, a context-specific network without updated GPRs would still have flux through the same metabolic reaction as Gene_A has not been properly removed. Thus, comparisons of genes between the four networks was ignored.

The scope of the Jerby and Gille networks is much greater than the HM (Figure 1). Reaction and metabolite count was about double. This is expected as the HM captures core hepatic metabolism while the Jerby and Gille networks try to capture all hepatic metabolism. It was interesting to see a great variation between all three networks, though the HM and Jerby networks were closer to each other than the Gille network when considering intracellular reactions. Both the HM and Gille incorporated additional network functionality outside of Recon 1, with some overlap. Additional functionalities are described in the main text. The Jerby network does not have additional functionalities as it is derived from Recon 1.

**References:**

1. Jerby L, Shlomi T, Ruppin E (2010) Computational reconstruction of tissue-specific metabolic models: application to human liver metabolism. Mol Syst Biol 6: 401.

2. Gille C, Bolling C, Hoppe A, Bulik S, Hoffmann S, et al. (2010) HepatoNet1: a comprehensive metabolic reconstruction of the human hepatocyte for the analysis of liver physiology. Mol Syst Biol 6: 411.


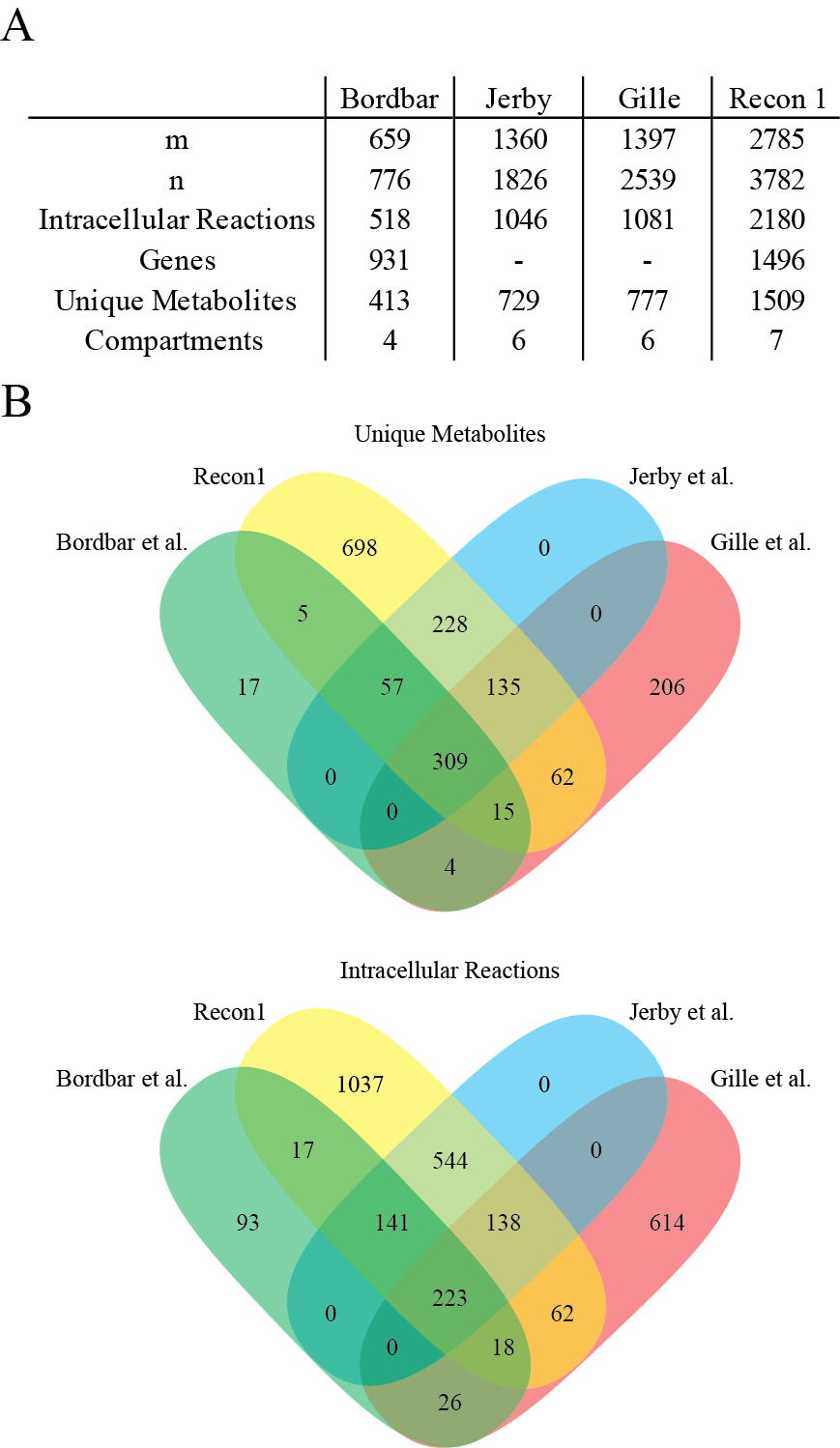


**Figure 1:** Metabolite and intracellular reaction comparison of the HM, two existing hepatic metabolic reconstructions, and the global metabolic network. The scope of the existing networks is much larger than the HM. The HM and Gille networks incorporate additional hepatic metabolic functionalities, outside of the scope of Recon 1.
